# FSAP aggravated endothelial dysfunction and neurological deficits in acute ischemic stroke due to large vessel occlusion

**DOI:** 10.1038/s41392-021-00802-1

**Published:** 2022-01-07

**Authors:** Dai-Shi Tian, Chuan Qin, Luo-Qi Zhou, Sheng Yang, Man Chen, Jun Xiao, Ke Shang, Dale B. Bosco, Long-Jun Wu, Wei Wang

**Affiliations:** 1 Department of Neurology, Tongji Hospital, Tongji Medical College, Huazhong University of Science and Technology, 430030 Wuhan, China; 2 Department of Neurology, Mayo Clinic, Rochester, MN 55905 USA

**Keywords:** Neuro-vascular interactions, Molecular neuroscience, Predictive markers, Neurological disorders

## Abstract

Revascularization and angiogenesis, as substrates of sustained collateral circulation, play a crucial role in determining the severity and clinical outcome of acute ischemic stroke (AIS) due to large vessel occlusion (LVO). Developing an adjunct biomarker to help identify and monitor collateral status would aid stroke diagnosis and prognosis. To screen the potential biomarkers, proteomic analysis was performed in this study to identify those distinct plasma protein profiles in AIS due to LVO with different collateral status. Interestingly, we found that levels of Plasma Factor VII Activating Protease (FSAP) significantly increased in those AIS patients with poor collaterals, and were correlated with worse neurological outcome. Furtherly, both in vitro and in vivo models of ischemic stroke were used to explore pathological mechanisms of FSAP in endothelial dysfunction. We demonstrated that the FSAP inhibitor, high-molecular-weight hyaluronan (HMW-HA), enhanced the pro-angiogenic vascular factors, improved the integrity of brain blood barrier, and promoted newly formed cerebral microvessels in the ischemic penumbra, consequently improving neurological function. To elucidate the pathways that might contribute to revascularization during LVO, we applied transcriptomic analysis via unbiased RNA sequencing and showed that Wnt signaling was highly involved in FSAP mediated endothelial dysfunction. Notably, inhibition of Wnt5a largely reversed the protective effects from HMW-HA treatment, implying that FSAP might aggravate endothelial dysfunction and neurological deficits by regulating Wnt5a signaling. Therefore, FSAP may represent a potential biomarker for collateral status after LVO and a promising therapeutic target to be explored in the treatment of stroke.

## Introduction

Approximately 40% of acute ischemic strokes are caused by proximal intracranial large vessel occlusions (LVO) that are also associated with poor clinical outcomes.^1,2^ Current management strategies for patients with ischemic stroke due to LVO include early reperfusion with intravenous tissue-type plasminogen activator (tPA) and/or intra-arterial thrombectomy.^3^ However, most stroke patients with LVO remain untreated because they diagnosed beyond the optimal time window for acute reperfusion therapies, or are unable to reach major stroke centers that are capable of thrombectomy.^1^

After arterial occlusion, alternative blood flow pathways, termed collaterals, can sustain viability of the penumbral brain regions for a period of time.^3^ Infarct growth varies substantially between individuals and is modulated by collateral blood flow^1,3^ and inflammation secondary to ischemia.^4–6^ Mounting clinical evidence suggests that collateral status is an independent predictor of outcome and response to recanalization therapies in patients with ischemic stroke.^7,8^ Additionally, maintaining good collaterals can limit infarct growth over time in persistent LVO.^1^ Angiogenesis involving proliferation of endothelial cells and formation of new vessels, also affects sustained collateral circulation and plays a crucial role in determining the outcome and severity of ischemic injury.^7^

Advanced neuroimaging modalities that are capable of providing both angiographic and perfusion information may help identify collateral status and thus guide therapeutic interventions.^7^ Digital subtraction angiography (DSA) is considered the gold standard for imaging collateral status.^9^ However, its invasive nature limits its applications. Conversely, the non-invasive nature of CT angiography makes it a good choice for assessing the collateral status in patients with acute stroke.^7^ However, non-contrast CT or magnetic resonance imaging (MRI)-based approaches to assess collateral status are lacking and require direct comparison with DSA.^10^ Additionally, access to multiple neuroimaging modalities is often restricted to major stroke centers and unavailable in many parts of the world, making it impossible to track dynamic changes of collateral circulation during the long term follow up after LVO. Consequently, a simple, fast, and sensitive technique for collateral monitor, such as a blood test, is of paramount importance.

As noted previously, identification and monitoring of molecular biomarkers in AIS would aid stroke diagnosis, prognosis, and development of therapeutic strategies.^11,12^ Some biomarkers were found related to collateral status,^13^ however, the incidence and underlying pathophysiology of these biomarkers remain poorly defined in the general population. In the present study, using proteomics and ELISA analysis, we screened differentially expressed proteins between patients with LVO-induced AIS but different collateral status. We demonstrate that plasma FSAP levels could indicate collateral status, and have potential prognostic value for neurological function at 3 months. We further show that FSAP can decrease collateral blood flow during the acute phase and inhibit collateral formation in experimental ischemic stroke. The signaling pathways involved were explored as high levels of FSAP could result in the disturbance of collaterogenesis and endothelium function. These findings are not only fundamentally important to understanding collateral formation, but also provide information needed to develop therapies to sustained collateral circulation in patients with persistent LVO.

## Results

### Plasma FSAP levels increased in patients with ischemic stroke and were correlated with 3-month outcome

**Fig. 1:**
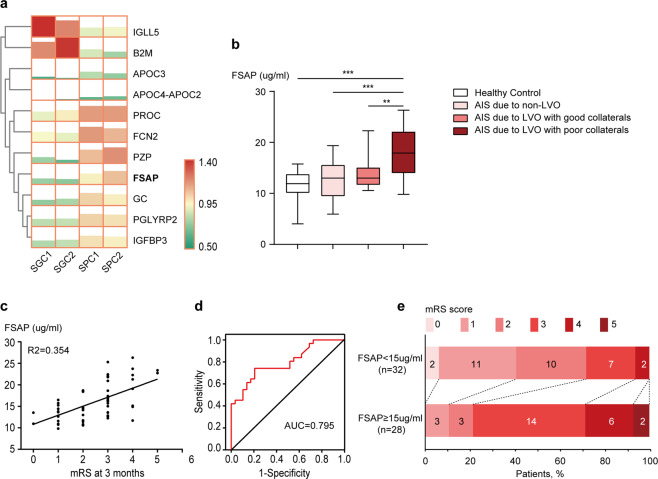
Plasma FSAP levels increased in patients with ischemic stroke and were correlated with 3-month outcome. **a** Heatmap representation of hierarchical clustering of differentially expressed proteins based on proteomic analysis. The scale from 0.5 green (low abundance) to 1.4 red (high abundance) represents normalized abundance in arbitrary units. **b** Validation of FSAP expression by ELISA analysis. Data is expressed as median ± interquartile ranges; *n* = 30 for each group. **p < 0.01, ***p < 0.001, horizontal lines and corresponding asterisks compare samples aligned with each end of the horizontal line by Kruskal–Wallis *H*-test. **c**–**e** Association of plasma FSAP levels with mRS 3 months post ischemia. Linear regression analysis of plasma FSAP levels with mRS 3 months post ischemia (**c**), receiver operating characteristic curve of plasma FSAP levels in prognosis of poor outcome (mRS 3–6) (**d**), and the distribution of the mRS scores at 3 months by FSAP levels (**e**)

Baseline characteristic details of the patient and control participants in the study are summarized in Supplementary Tables 1–2. According to the ASITN/SIR Collateral Flow Grading System, 40 patients with AIS due to LVO were divided into two groups: AIS with good collaterals (ASTIN/SIR 3–4), and AIS with poor collaterals (ASTIN/SIR 0–2). There were no significant differences in age, sex, National Institute of Health Stroke Scale (NIHSS) score at baseline, medical history, or laboratory data between the different groups with AIS. Plasma samples were analyzed by proteomics technique (Supplementary Figs. 1, 2) and eleven differentially expressed proteins were finally acquired with a 1.2-fold/0.83-fold or greater difference in abundance in AIS with poor collaterals compared to those with good collaterals (Fig. 1a, and Supplementary Table 3). Among these proteins, FSAP is known to be involved in blood hemostasis and endothelial function. ^14,15^ We then verified the differential abundance of FSAP using ELISA analysis. Consistent with the tendency shown during proteomic analysis, ELISA results also presented similar alterations in an independent population. For instance, the levels of FSAP were significantly higher in poor-collateral group compared to other groups, including the healthy control, AIS due to non-LVO, and AIS with good collaterals group (Fig. 1b). Plasma FSAP levels slightly increased in patients with stroke due to non-LVO and AIS with good collaterals compared to healthy controls.

We further investigated the association between plasma FSAP levels and prognosis of AIS due to LVO. A positive correlation was found between FSAP protein levels and the modified Rankin scale (mRS), a measure of neurological outcome, at a 3-month follow-up (*R*^2^ = 0.354 and *p* < 0.001; Fig. 1c). Patients were divided into those with good prognosis (mRS 0–2) and those with poor prognosis (mRS 3–6). Logistic analysis results showed that higher FSAP levels (per 1 µg/ml) predicted poor outcomes (odds ratio 1.399, 95% CI 1.123–1.744, *p* = 0.003, after adjusting for age, sex, and NIHSS at onset). The areas under the receiver operating characteristic (ROC) curve (AUC) were 0.795 (0.684–0.907) for distinguishing patients with poor prognosis from patients with good prognosis, which was interpreted as a good classification performance (Fig. 1d). Twenty-eight patients with acute LVO were further allocated to the elevated FSAP level group (≥15 µg/ml), and the other 32 were allocated to lower FSAP level group (<15 µg/ml). An ordinal comparison of the distribution of patients across mRS categories at 3 months demonstrated that lower FSAP levels (<15 µg/ml) were negatively related with poorer outcomes in those survivors of ischemic stroke (common odds ratio 0.128, 95% CI 0.045–0.370; *p* < 0.001; Fig. 1e).

### Pathological expression of FSAP and vascular factors in experimental models of ischemic stroke

To test the translational relevance of our clinical findings, we used primary mBMECs exposed to hypoxia and reoxygenation, and a murine model of ischemic stroke MCAO. We first measured FSAP expression in primary mBMEC cultures subjected to 2–8 h OGD and 24 h reoxygenation. Western blot data revealed that OGD/reoxygenation significantly increased the expression of FSAP (Supplementary Fig. 3a). We then analyzed the expression of FSAP within the plasma and brains of mice following MCAO. FSAP level in brain remained significantly elevated over 28 days of post MCAO when compared with controls, peaking 3 days after ischemia (Supplementary Fig. 4a). Similarly, plasma FSAP expression was significantly upregulated following MCAO when compared to controls, also peaking 3 days after ischemia, but returned to normal levels after 28 days (Supplementary Fig. S4c).

To compare the time course of FSAP expression to induction of angiogenesis, we analyzed the protein expression of several proangiogenic factors in the penumbra following MCAO. Stroke induced a significant increase in VEGFA, VEGFR2, FGF2, and FGFR1 expression 1–3 days after MCAO. A significant decline occurred 7 days after ischemia, except for FGFR1 which peaked at 7 days, and gradually declined to normal by 28 days (Supplementary Fig. 4f, g). Meanwhile, stroke induced a significant decrease in Claudin5 and ZO-1 expression 1–3 days after MCAO, and then gradually increased until 28 days, demonstrating the temporal changes that occur to the integrity of the brain blood barrier (BBB) (supplementary Fig. 4f, g). Interestingly, FSAP exhibited the highest expression level around 3 days after MCAO, implicating a negative correlation between FSAP levels and the investigated angiogenic factors and BBB leakage post ischemic insults.

### Suppression of FSAP improves sensorimotor and cognitive functions, and attenuates neuronal loss and infarcted volume after cerebral ischemia

**Fig. 2:**
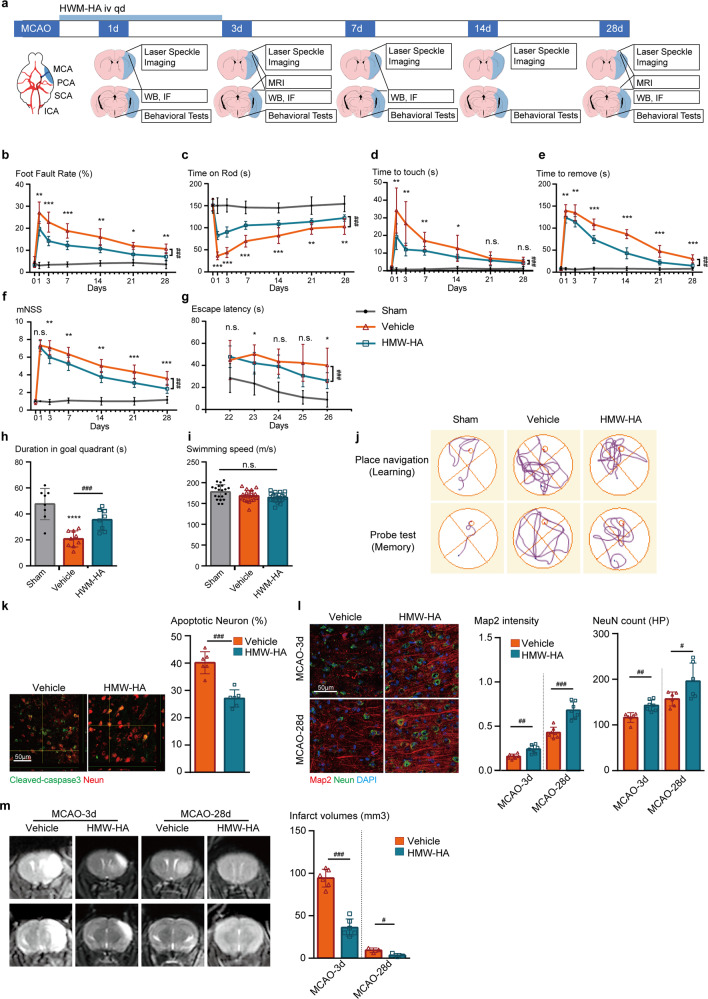
Suppression of FSAP by HMW-HA treatment improved sensorimotor and cognitive outcomes, and attenuates brain infarction after cerebral ischemia. **a** Schematic diagram of the experimental design. **b**–**f** Sensorimotor deficits were evaluated 1 days before and up to 28 days after MCAO by foot fault test (**b**), rota-rod test (**c**), adhesive tape removal test (**d**), time to touch the tape and (**e**), time to remove the tape, and **f** the modified neurological severity score (mNSS). Data is expressed as mean ± SD; *n* = 6–12 for each group; *p < 0.05, **p < 0.01, ***p < 0.001 versus Vehicle group by one-way ANOVA followed by Tukey’s multiple comparison tests (individual time point); ###*p* < 0.001 between vehicle and HMW-HA groups by two-way ANOVA with repeated analysis. **g**–**j** Long-term cognitive functions were assessed by the Morris water maze. **g** The time for the animals to locate the submerged platform (escape latency) was measured at 22–26 days after MCAO. *p* < 0.05, **p < 0.01, ***p < 0.001 versus vehicle group by one-way ANOVA followed by Tukey’s multiple comparison tests (individual time point); ###*p* < 0.001 between vehicle and HMW-HA groups by two-way ANOVA with repeated analysis. **h** Spatial memory was evaluated at 27 days after MCAO by measuring the time spent in the goal quadrant when the platform was removed. ****p < 0.0001, vs. Sham; ###*p* < 0.001, horizontal lines and corresponding asterisks compare samples aligned with each end of the horizontal line by two-way ANOVA followed by Bonferroni’s multiple comparison tests. **i** Gross locomotor function was measured by average swim speed. **j** Representative swim path for different experimental groups. Data for each experiment is expressed as mean ± SD; *n* = 9–12 for each group. **k**–**m** Neuron apoptosis, neuron loss and brain infarction were measured and quantified by immunostaining and MR imaging. Representative (**k**) cleaved-caspase 3 (green) and NeuN (red) immunofluorescent images with Z-section of confocal analysis, **l** representative NeuN (green) and Map2 (red) immunofluorescent images, and **m** representative MR images with accompanying quantitative analysis of vehicle and HMW-HA groups at 3 and 28 days after MCAO. Data is expressed as mean ± SD; *n* = 3–7 for each group. *p < 0.05, **p < 0.01, ***p < 0.001, ****p < 0.0001 vs. vehicle group by one-way ANOVA followed by Tukey’s multiple comparison tests

In line with the time course of FSAP expression and its function, we administered the FSAP inhibitor HMW-HA for three days immediately following stroke induction. A schematic diagram of the experimental design is given in Fig. 2a. Administration of HMW-HA effectively inhibited the high levels of FSAP induced by MCAO, in both ischemic brain tissue and plasma (Supplementary Fig. 4c, d).

To assess neurological deficits after ischemic damage, we utilized a battery of behavioral evaluations to comprehensively examine sensorimotor functions during 28 days after MCAO (Fig. 2b–f). Sensorimotor function deficits were shown in all behavioral tests (foot fault, rotarod, and adhesive tape removal tests), and were especially prominent over the first week post MCAO. Interestingly, administration of HMW-HA significantly improved sensorimotor deficits and neurological recovery when compared to vehicle group in all the four behavioral tests at different time points, as evidenced by statistically significant fewer foot faults (1–28 days after MCAO), longer time on the rotarod (1–28 days after MCAO), quicker response time to touch (1–14 days after MCAO) and to remove (1–28 days after MCAO) the adhesive tape from the contralateral forepaw, and lower mNSS scores (3–28 days after MCAO) (Fig. 2b–f).

We then performed Morris water maze evaluation to assess long-term spatial cognitive decline in mice after MCAO (Fig. 2g–j). Compared to vehicle controls, long-term learning and memory behavior deficits were improved by HMW-HA treatment after MCAO, as shown by the significant decrease in escape latency during the learning phase (Fig. 2g) and increased time spent in the goal quadrant during the memory phase (Fig. 2h). Swimming speed between different groups was comparable (Fig. 2i), eliminating speed as a confounding factor in assessing time-based outcomes during MCAO.

To explore the effects of HMW-HA administration on neuronal injury after stroke, the intensity and number of cleaved-caspase 3 and microtubule-associated protein 2 (MAP2) positive neurons were determined via immunostaining (Supplementary Fig. 5 and Fig. 2k, l). We found lower proportion of neuron apoptosis, more surviving neurons, and less loss of MAP2 3 days and 28 days after MCAO in the HMW-HA group when compared to vehicle controls. In addition, HMW-HA treatment significantly reduced brain infarct volume as determined by MR imaging at and 28 days after MCAO, and improved overall animal survival (Supplementary Fig. 6).

Taken together, these data indicated that inhibition of FSAP improves stroke outcome and recovery both at the histological and neurobehavioral levels.

### Suppression of FSAP enhances revascularization, improves the integrity of the BBB, and promotes newly formed microvessels in the penumbral area of ischemic brains

**Fig. 3:**
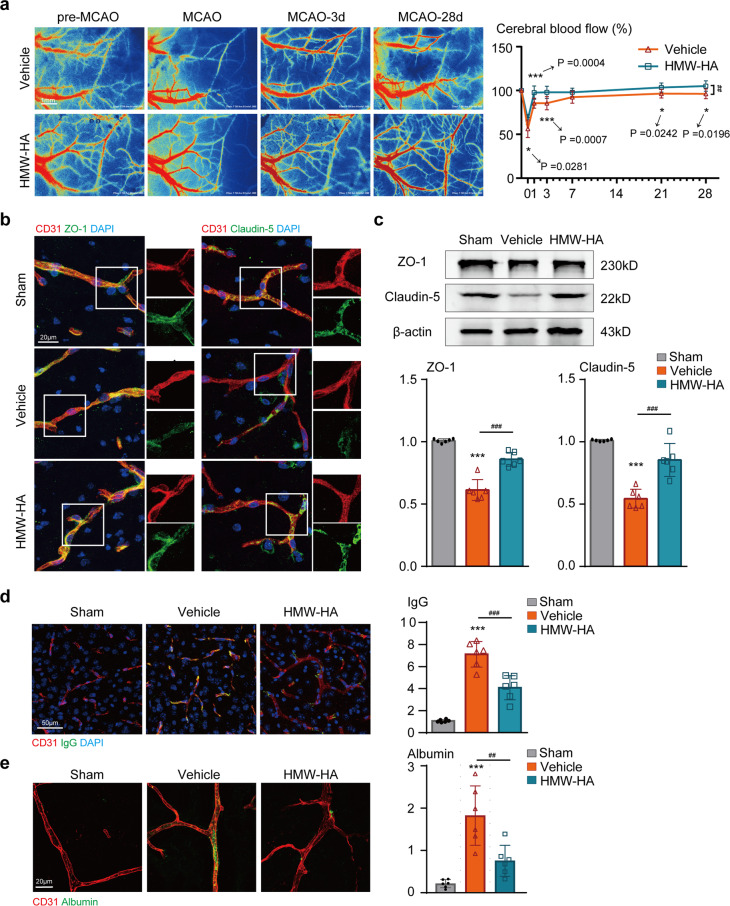
Suppression of FSAP by HMW-HA treatment improves cerebral blood flow recovery in mice after cerebral ischemia. **a** Representative CBF images were shown at pre-MCAO, and 15 min, 3, 28 days after MCAO. Quantitative analysis of the relative CBF which was first determined as the ratio of ischemic to non-ischemic values, and then normalized to the pre-MCAO baseline for each animal. Data is expressed as mean ± SD; *n* = 6–12 for each group; *p < 0.05, ***p < 0.001 versus vehicle group by one-way ANOVA followed by Tukey’s multiple comparison tests (individual time point); ##*p* < 0.01 between vehicle and HMW-HA groups by two-way ANOVA with repeated analysis. **b**, **c** Blood-brain barrier structure was measured and quantified by immunostaining and Western blot. **b** Representative ZO-1 (green) and CD31 (red) immunofluorescent images, representative Claudin-5 (green) and CD31 (red) immunofluorescent images, and **c** representative western blot images and quantitative analysis in vehicle group and HMW-HA group at 3 days following MCAO. Data is expressed as mean ± SD; *n* = 6 for each group; ***p < 0.001 versus Sham group, ###*p* < 0.001 versus vehicle group by one-way ANOVA followed by Tukey’s multiple comparison tests. **d**, **e** Blood-brain barrier leakage was detected and quantified by extravasated IgG or albumin (green) and CD31 (red) immunostaining in the penumbral regions at 3 days after MCAO. Representative immunostaining images and quantitative analysis of IgG intensity (**d**) and albumin (**e**). Data is expressed as mean ± SD; *n* = 6 for each group; ***p < 0.001 versus Sham group, ###*p* < 0.001, ##*p* < 0.01 versus Vehicle group by one-way ANOVA followed by Tukey’s multiple comparison tests

On the basis of the improvement in neurological outcomes during HMW-HA administration and the close correlation between FSAP and collaterals, we speculated that suppression of FSAP might increase collateral blood flow within the penumbral area during stroke recovery. To this end, we measured the spatiotemporal changes of ischemic cortical cerebral blood flow (CBF) using the laser speckle imaging. HMW-HA treatment considerably improved CBF recovery from 15 min during MCAO to 28 days reperfusion after MCAO, when compared to Vehicle controls (Fig. 3a). These results were consistent with better long-term neurological recovery in HMW-HA-treated mice after stroke as described above.

To assess the disruption of structural integrity and paracellular permeability of the BBB, we examined the expression and distribution of tight junction (TJ)-associated proteins, Claudin 5 and ZO-1, in the penumbral area of ischemic brains, and then quantified the IgG leakage into brain tissue sections 3 days after MCAO. We found that the expression levels and localization of these TJ proteins (Claudin5 and ZO-1) were greatly reduced in vehicle-treated mice, and that HMW-HA administration could largely reverse this disruption (Fig. 3b, c). Moreover, the IgG leakage and extravasated albumin were significantly decreased in HMW-HA treated mice when compared to Vehicle group (Fig. 3d, e), suggesting that inhibition of FSAP could stabilize BBB integrity during MCAO.

**Fig. 4:**
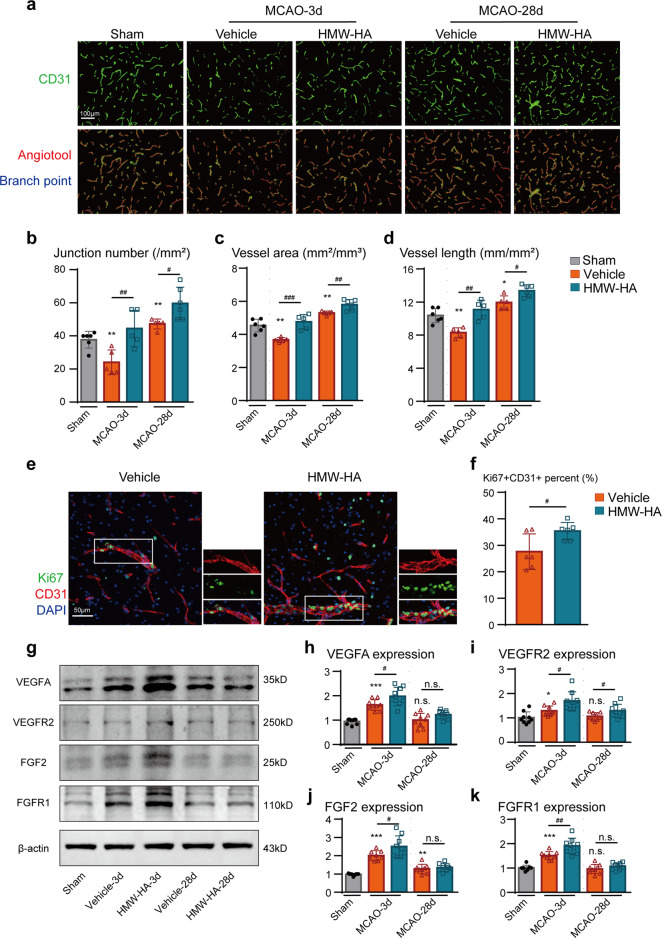
Suppression of FSAP by HMW-HA treatment enhances revascularization and newly formed microvessels in the penumbral regions of mouse brains after cerebral ischemia. **a**–**d** Cerebral vasculature was detected and quantified by CD31 (green) immunostaining in the penumbral regions at 3 and 28 days after MCAO. **a** Representative CD31 immunostaining images, **b** quantitative analysis of junction number, **c** vessel area, and **d** vascular length. Data is expressed as mean ± SD; *n* = 5–7 for each group. *p < 0.05, **p < 0.01 versus Sham group, #*p* < 0.05, ##*p* < 0.01, ###*p* < 0.001 versus vehicle group by one-way ANOVA followed by Tukey’s multiple comparison tests. **e**, **f** CD31 (red) and Ki67 (green) double-immunostaining was used to determine the newly formed microvessels in the penumbral regions of the brains at 3 days after MCAO. **e** Representative images, and **f** quantification of the Ki67+/CD31+ signals. Scale bar, 50 μm. Data is expressed as mean ± SD; *n* = 5–7 for each group; #*p* < 0.05 versus vehicle group by Student’s *T*-test. **g**–**k** Pro-angiogenic factors expression was detected by Western blot. **g** Representative western blot images and **h**–**k** quantitative analysis. Data is expressed as mean ± SD; *n* = 9 for each group. n.s. not significant, *p < 0.05, ***p < 0.001 versus Sham group, n.s. not significant, #*p* < 0.05, ##*p* < 0.01 versus vehicle group by one-way ANOVA followed by Tukey’s multiple comparison tests

We further used CD31 immunostaining to identify the changing microvessels. As illustrated in Fig. 4a–d, more CD31 positive microvessels were present in HMW-HA treated ischemic brains than vehicle controls 3 and 28 days after MCAO. More newly formed microvessels were identified as CD31 and Ki67 double positive in HMW-HA treated mice 3 days after MCAO (Fig. 4e, f). Western blot analysis confirmed increased post-stroke cerebral angiogenesis in HMW-HA treated mice, as presented by higher expression of VEGFR, VEGF2, FGF2, and FGFR1 3 days following MCAO (Fig. 4g, k).

These findings suggested that inhibition of FSAP increases the integrity of the BBB, enhancing revascularization and promoting new microvessel growth, resulting in vascular remodeling and sustained collateral blood flow after ischemic stroke.

### FSAP impairs BBB integrity and angiogenesis in cultures of mBMVECs

**Fig. 5:**
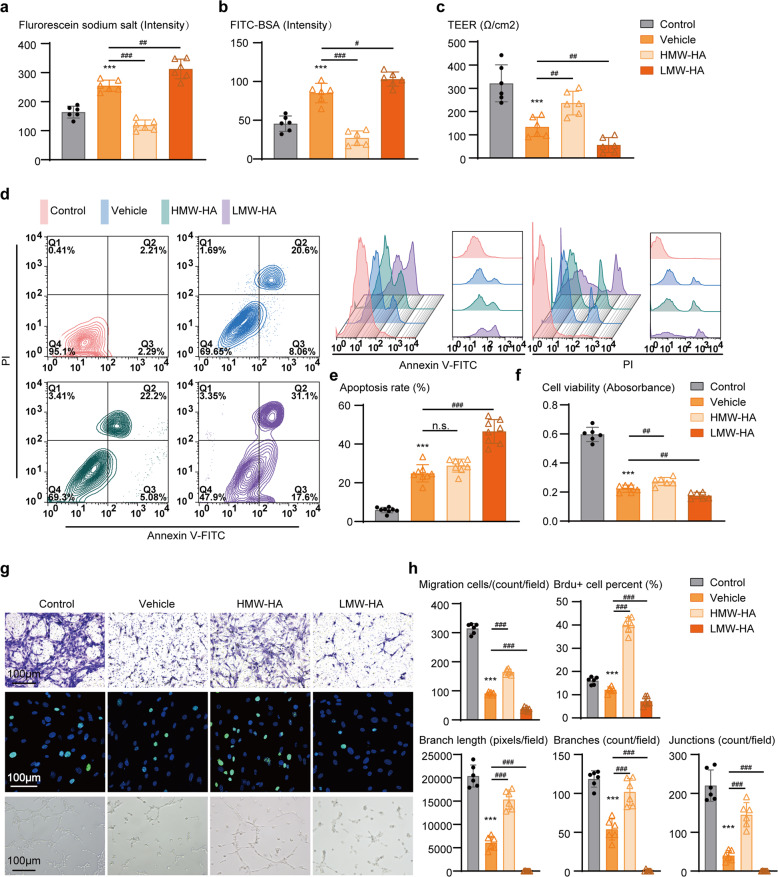
Suppression and stimulation of FSAP impairs or promotes BBB integrity and angiogenesis in cultures of mouse brain microvascular endothelial cells (mBMECs) after OGD, respectively. **a**–**c** The paracellular permeability and Trans Endothelial Electrical Resistance (TEER) value were measured to reflect the barrier property of the mBMECs monolayer after OGD. **a** Quantitative analysis of the intensity of infiltrated fluorescein sodium, **b** FITC-BSA, and **c** monolayer electrical resistance. Data is expressed as mean ± SD; *n* = 6 for each group; ***p < 0.001 versus control group, #*p* < 0.05, ##*p* < 0.01, ###*p* < 0.001 versus vehicle group by one-way ANOVA followed by Tukey’s multiple comparison tests. **d**–**f** Apoptotic cells were stained with annexin V-FITC and propidium iodide and analyzed using a FACS Calibur flow cytometer, and cell viability was measured by Cell Counting Kit-8. **d** Representative images of FACS analysis of apoptotic cells, **e** quantitative analysis of apoptotic rates, and **f** cell viability. Data is expressed as mean ± SD; *n* = 6 for each group; ***p < 0.001 versus control group, n.s. not significant, ##*p* < 0.01, ###*p* < 0.001 versus vehicle group by one-way ANOVA followed by Tukey’s multiple comparison tests. **g** Representative images of transwell migration assays, BrdU incorporation assays, and tube formation assays, and **h** quantitative analysis of migration cells, proliferative cells, branch length, branch counts, and junction counts. Data is expressed as mean ± SD; *n* = 6 for each group; ***p < 0.001 versus Control group, ###*p* < 0.001 versus Vehicle group by one-way ANOVA followed by Tukey’s multiple comparison tests

To investigate the direct effects of FSAP on endothelium function, we harvested primary endothelial cells from mouse brain tissue to study in an OGD model. LMW-HA and HMW-HA were administered to upregulate and downregulate FSAP expression (Supplementary Fig. 3d). We assessed the barrier function using monolayers of primary endothelial cells. In accordance with the in vivo results, we observed significantly stronger fluorescence intensities of both fluorescein sodium and FITC-BSA, and decreased TEER values in OGD model, suggesting the damage in BBB integrity in vitro. HMW-HA treatment decreased the fluorescence intensities of both fluorescein sodium and FITC-BSA and increase TEER values, indicating a tighter BBB due to suppression of FSAP (Fig. 5a–c). However, as upregulation of FSAP by LMW-HA showed a directly opposed response regarding the fluorescein sodium, FITC-BSA and TEER values, we suggest that FSAP could be a relevant factor in regulating barrier integrity in brain endothelium.

Additionally, we examined the relevance between hypoxia-induced FSAP and endothelial cell viability and apoptosis. The results showed that suppression of FSAP by HMW-HA increased endothelial viability, and activation of FSAP by LMW-HA accelerated endothelial apoptosis and decreased cell viability (Fig. 5d–f).

We further examined the effects of FSAP on pro-angiogenic activities using transwell migration assays with mBMECs. FSAP inhibition by HMW-HA led to significantly increased endothelial cell migration, whereas upregulation of FSAP by HMW-HA exerted the opposite effect (Fig. 5g, h). In vitro angiogenesis was also measured by BrdU proliferation assay, since cell proliferation actively participates in angiogenesis. Compared to Vehicle controls, HMW-HA treatment upregulated cell proliferation in mBMECs, while LMW-HA treatment suppressed the proliferation (Fig. 5g, h). Moreover, capillary tube formation assays demonstrated that suppression of FSAP in mBMECs significantly increased tube formation, showing elevated branch length, branch number, and junction count (Fig. 5g, h). In contrast, upregulation of FSAP by LMW-HA significantly decreased tube formation, showing reduced branch length, branch number, and junction count. Collectively, these results demonstrate that FSAP disrupts BBB integrity, promotes hypoxia induced endothelial apoptosis, and inhibits endogenous angiogenic activities in mBMECs following OGD.

### Transcriptomic signature of FSAP inhibition on endothelium exposed to OGD

**Fig. 6:**
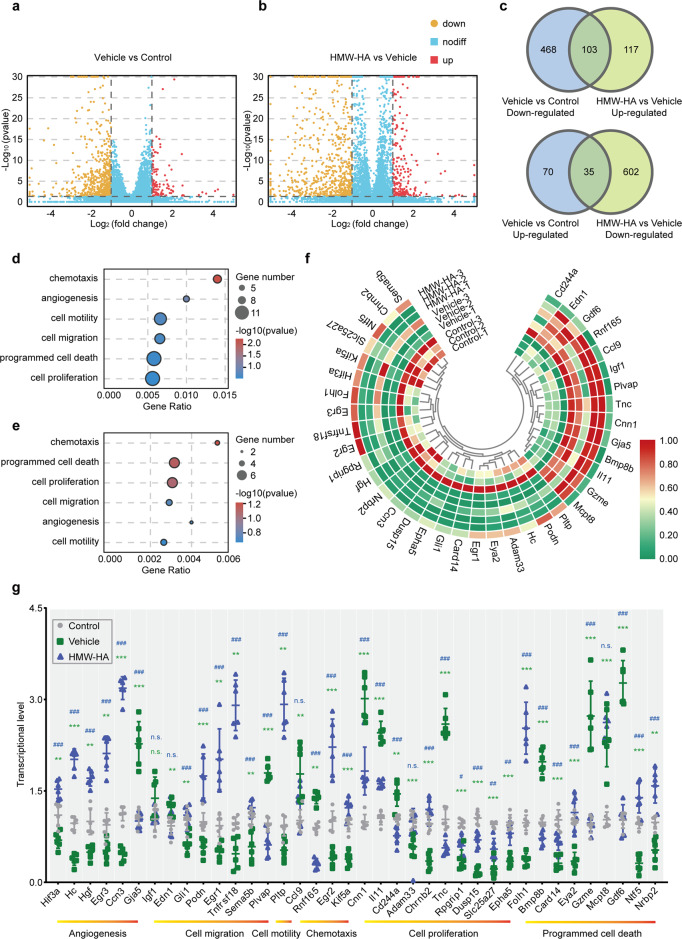
Endothelial transcriptional changes following OGD and HMW-HA treatment. **a**, **b** The volcano plot for differentially genes (DEGs) expression. The *x*-axis represents the log2 (fold change), the *y*-axis represents the log10 (*p* value) of the repeated test results. The red region contains the upregulated genes, and the green region contains the downregulated ones. Three biological replicates were used for RNA sequencing analysis. **c** Venn diagrams of the numbers of upregulated and downregulated gene changes in the endothelial cells observed following OGD with or without HMW-HA treatment, depicting the overlap of changes found in different groups. **d**, **e** GO term enrichment analysis of DEGs between **d** control and vehicle group and between **e** vehicle and HWA-HA group. The six biological process GO terms related to angiogenesis process was shown. The *y*-axis represents GO biological terms. The *X*-axis represents Gene ratio. The size of the dot represents the number of genes under a specific term. The color of the dots represents the adjusted *p*-value. **f** The Heatmap image depicting transcriptional changes of the 38 selected genes in different groups. **g** Validation of transcriptional changes of the selected 38 genes using RT-qPCR analysis. Data is expressed as mean ± SD; *n* = 6 group; n.s. not significant, **p < 0.01, ***p < 0.001 versus control group, n.s. not significant, #*p* < 0.05, ##*p* < 0.01, ###*p* < 0.001 versus vehicle group by one-way ANOVA followed by Tukey’s multiple comparison tests

To further our understanding of the dysregulated state in OGD-exposed and FSAP mediated endothelial dysfunction, transcriptomic analysis via unbiased RNA sequencing was performed. Volcano plots showed genes in the endothelium that were upregulated or downregulated by more than two-fold in OGD when compared to controls, and in HMM-HA-treated and vehicle-treated OGD-exposed endotheliums (Fig. 6a, b). Venn diagrams summarized the number of differentially expressed genes (DEGs) as well as the intersection of genes regulated contrarily in the pairwise comparison (Fig. 6c).

Next, we performed Gene Ontology (GO) term analysis for the DEGs in each comparison. GO term pathways in this endothelial dysfunction module included those pathways involved in chemotaxis, angiogenesis, cell motility, migration, programmed cell death, and proliferation, highlighting a role for viability, angiogenesis, and reconstruction in endothelial dysfunction (Fig. 6d, e). Candidate transcripts were evaluated and selected based on GO term analysis and through existing literature describing endothelial function. The 38 identified genes that changed exclusively in each pairwise comparison were given in Supplementary Table 4 and Supplementary Fig. 7. Heatmaps showed the differentially expressed level of the 38 identified transcripts (Fig. 6f), which were then validated by quantitative PCR **(**Fig. 6g). In general, OGD exposure inhibited endothelial genes that related to angiogenesis, migration, motility, chemotaxis, proliferation, and induced programmed cell death. HMW-HA, the inhibitor of FSAP, largely reversed these effects (Fig. 6f, g). These results suggested that FSAP might aggravate OGD-induced endothelial dysfunction, and inhibition of FSAP could protect endothelium function from hypoxia.

### FSAP regulates endothelial function by Wnt5a signaling

**Fig. 7:**
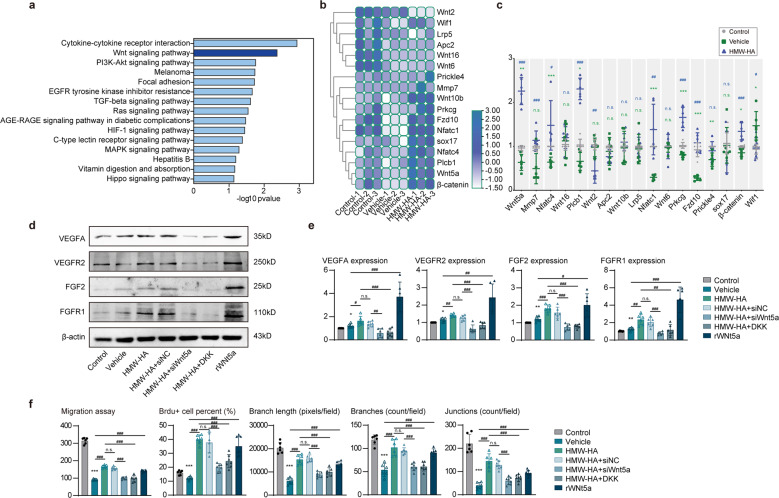
FSAP regulates endothelial function by Wnt5a signaling. **a** The top 15 KEGG enriched pathways of the 38 selected genes. **b**, **c** The Heatmap image depicting transcriptional changes of the members of Wnt signaling pathway in different (**b**) groups, and **c** RT-qPCR validations. Data is expressed as mean ± SD; *n* = 5–7 for each group; n.s. not significant, **p < 0.01, ***p < 0.001 versus Control group, n.s. not significant, #*p* < 0.05, ##*p* < 0.01, ###*p* < 0.001 versus vehicle group by one-way ANOVA followed by Tukey’s multiple comparison tests. **d**, **e** Pro-angiogenic factors expression was detected by Western blot. **d** Representative Western blot images and **e** quantitative analysis. Data is expressed as mean ± SD; *n* = 6–8 for each group; n.s. not significant, *p < 0.05, ***p < 0.001 versus control group, n.s. not significant, #*p* < 0.05, ##*p* < 0.01, horizontal lines and corresponding hashes compare samples aligned with each end of the horizontal line by one-way ANOVA followed by Tukey’s multiple comparison tests. **f** Quantitative analysis of migration cells, proliferative cells, branch length, branch counts, and junction counts. Data is expressed as mean ± SD; *n* = 6–8 for each group; n.s. not significant, *p < 0.05, ***p < 0.001 versus Control group, n.s. not significant, #*p* < 0.05, ##*p* < 0.01, horizontal lines and corresponding hashes compare samples aligned with each end of the horizontal line by one-way ANOVA followed by Tukey’s multiple comparison tests

Kyoto Encyclopedia of Genes and Genomes (KEGG) pathway analysis showed that Wnt signaling was highly involved in FSAP mediated endothelial dysfunction (Fig. 7a). In addition, the Wnt pathway has been identified as a key regulator of CNS angiogenesis, vascular formation and BBB maintenance.^16,17^ Thus, we evaluated the expression of Wnt pathway members by quantitative PCR. Our results showed that mRNA levels of Wnt5a were significantly elevated by HMW-HA in OGD-exposed mBMVECs, whereas the levels of Wnt2, Wnt6, Wnt10b, and Wnt16 did not change significantly (Fig. 7b, c).

To understand the function of Wnt5a in FSAP related endothelial dysfunction, we used DKK, a Wnt5a inhibitor and siRNA to downregulate Wnt5a expression. The results showed that HMW-HA treatment increased the expression level of some proangiogenic factors, including VEGFR, VEGF2, FGF2, and FGFR1, in OGD-exposed mBMVECs (Fig. 7d, e). However, inhibition of Wnt5a pathway by DKK or Wnt5a knockdown reversed this effect. In addition, the pro-angiogenic activities, including endothelial migration, proliferation, and tube formation induced by the FSAP inhibitor, were largely abolished by Wnt5a inhibition or knockdown (Fig. 7f).

**Fig. 8:**
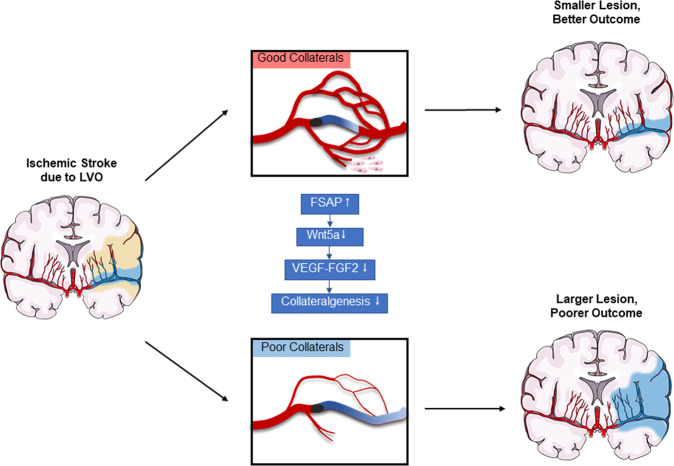
Take home figure. Plasma FSAP levels significantly increased in patients with AIS due to LVO and poor collaterals, and correlates with neurological outcome. Pathological expression of FSAP was validated in an experimental stroke model, and negatively associated with pro-angiogenic vascular factors. Suppression of FSAP enhances revascularization, improves the integrity of brain blood barrier, and promotes newly formed microvessels in the penumbral area of ischemic brains, and consequently decreased infarct size and neurological deficit. Mechanistic analysis revealed FSAP might facilitate endothelial dysfunction by regulating Wnt5a signaling

Collectively, these results demonstrated that the increase in FSAP expression following stroke aggravated endothelial dysfunction, resulted in poor angiogenesis and collaterals, and thus worsened neurological outcome. FSAP inhibition exhibited powerful proangiogenic and neuroprotective effects via Wnt5a activation, and strongly support our hypothesis that FSAP plays a key role in the dysregulation of collateral circulation after AIS due to LVO (Fig. 8).

## Discussion

This study identifies plasma Factor VII activating protease (FSAP) as a biomarker of collateral circulation after AIS due to LVO, and investigated the underlying mechanisms. Our main findings were: (1) LVO-induced AIS patient data strongly correlated collateral status with plasma FSAP levels, (2) FSAP levels were also able to independently predict clinical outcome at 3-month follow-ups, (3) FSAP could induce endothelium apoptosis, and weaken cell viability, proliferation, migration and tube formation of PBMECs, (4) FSAP blockade increased cerebral blood flow, improved BBB integrity, promoted collateral formation, and resulted in better neurological function in experimental stroke models, finally (5) FSAP modulated endothelium function via the Wnt5a pathway. Together, these findings highlight the potential utility of plasma FSAP as a biomarker and therapeutic target for collateral circulation in patients with AIS due to LVO.

Collateral blood flow, the potential vigorous network of vessels has been proven to be one of key determinants in clinical outcome of ischemic stroke patients,^3,8^ by providing perfusion to the penumbral region and reducing the progression of infarct size during reperfusion therapies. Moreover, whether receiving acute reperfusion therapies or not, the rate of infarct growth after LVO depends on the capacity to sustain collateral blood flow above thresholds of infarction over time.^1^ Currently, collateral circulation is usually assessed by neuroimaging. However, there is no ideal imaging modality for an accurate and dynamic measurement of the collateral flow and structure, as each method possesses various limitations.^7^ DSA is restricted by its invasive nature. CTA and MRA could reliably diagnose LVO and monitor collateral status, but still require evaluation at a hospital with angiographic imaging capabilities. Due to the ease of acquisition and the potential to summate vessel injury, thrombotic and angiogenesis information that relate to collateral blood flow, a blood-based biomarker could aid in monitoring collateral status and predicting outcome after stroke.

Most biomarkers of stroke have been identified on the basis of underlying pathophysiological processes, such as inflammation, coagulation, fibrinolysis, and brain tissue damage.^4,11^ Advances in technology are enabling use of high-throughput techniques to avoid selection bias and are generating extensive lists of molecules for evaluation as biomarkers. Among these omics-based approaches, proteomics remains the most useful tool for discovering new biomarkers.^11^ In the current study, we used proteomics to screen the biomarkers reflecting different collateral status in AIS due to LVO, and Western blots for validation. Eleven differentially expressed proteins were finally acquired with a difference in abundance in AIS with poor collaterals compared to those with good collaterals. Among them, FSAP has been shown to be involved in endothelial function,^14,15^ implicating that it might play a pivotal role in collateral status. We found plasma FSAP levels increase significantly as a result of AIS due to LVO within 7 days, especially in those with poor collateral and a slight increase in AIS due to small artery occlusion and LVO with good collaterals, when compared to healthy controls.

FSAP, encoded by hyaluronic acid binding protein 2 (HABP2), is a circulating plasma serine protease.^18^ Genetic epidemiological studies have shown that the single nucleotide polymorphism in/near the HABP2 gene are associated with advanced carotid stenosis and stroke.^19–21^ It has also been reported that the increase in FSAP level was found in patients with ischemic stroke, and focused chiefly on its prothrombotic function.^22^ In addition to activating factor VII, FSAP can also activate pro-urokinase as well as inactivate tissue factor pathway inhibitor,^23^ indicating a role in coagulation and fibrinolysis. Lower FSAP plasma levels were also implicated a higher chance of arterial recanalization after tissue plasminogen activator treatment.^24^ Our study, on the other hand, indicates that increases in FSAP levels was associated with poor collaterals in persistent LVO. Therefore, detection of FSAP level might provide potentially clinical hints in monitoring collateral status and predicting outcome after AIS.

Mechanisms linking FASP level and endothelial function as well as collateral status warrants further investigation. In experimental stroke models, our results indicate that FSAP expression was up-regulated following ischemia, peaking at 3 days, and maintained high-persistent level in both plasma and brain tissue within 1 month. Various angiogenic factors, such as VEGFA and FGF2, were shown to play a central role in the post-stroke vascular reconstruction.^25^ Suppression of FSAP at early stage robustly increased levels of VEGFA, FGF2, and their receptors VEGFR2 and FGFR1 in the mouse brain after stroke. Angiogenesis at late stage, sustained collateral circulation after stroke, determining the outcome and severity of ischemic injury, especially for those patients with large vessel occlusion.^7^ Suppression of FSAP enhanced angiogenesis and facilitated restoring cerebral blood supply. Consequently, FSAP inhibition increased focal blood flow at acute stage, improved the integrity of BBB, and higher cerebral vessel density at late stage, resulting in more survived neurons, smaller ischemic lesions, and ultimately better outcome. It was reported that, the lack of endogenous FSAP, in FSAP knockout mice, led to a poor outcome after MCAO, and this was associated with higher inflammation and lower cell survival due to dysfunction in astrocytes and neurons.^26^ This discrepancy can be reconciled considering FSAP may have multiple effects on different cell types. In addition, it is well known that the acute pharmacological inhibition is likely different from permanent genetic deficiency. After MCAO, both plasma and brain FSAP levels significantly elevated and peaked at 3 days after ischemic injury. Our results showed that early administration of FSAP inhibitor, HMW-HA, could downregulate FSAP to a relatively normal level, and protect brain lesions from aggravation of endothelial dysfunction by excessive FSAP after stroke onset.

We also addressed the molecular basis underlying FSAP-induced endothelial reprogramming. In addition to negatively regulate angiogenic factors and vascular integrity,^15^ our in vitro study showed that FSAP could modulate endothelium with multifunctional properties. The cellular effects of FSAP range from impairing endothelium survival, viability, and permeability, to inhibiting cell proliferation, migration, and tube formation. Again, using high-throughput technique, transcriptomics showed that FSAP might dysregulate endothelial function via Wnt pathway. Wnt5a signaling is a key regulator of CNS angiogenesis and BBB formation and maintenance, and has been specifically linked to the induction of solute transporters, tight junction proteins, efflux transporters and inhibition of transcytosis.^16,17,27^ In this study, we found that hypoxia could induce the production of FSAP and suppress Wnt5a/pathway, whereas inhibition of FSAP resulted in Wnt5a activation. Reversely, down-regulation of Wnt5a was sufficient to counteract endothelial protection of the FSAP inhibitor, suggesting that this signal pathway is a key factor in FSAP mediated altering brain endothelial gene expression during ischemic stroke and important for development and maintenance of proper collateral circulation.

Despite the novelty of screened biomarker and translational value in ischemic stroke, there is several limitations in the current study. First, the study was mostly restricted to a single race/ethnicity. Second, as blood samples were acquired beyond the typical treatment windows for reperfusion therapy, the prognostic ability of FSAP in hyperacute stroke remains unclear. The effects of vessel recanalization on plasma FSAP levels, and whether FSAP levels could reflect collateral status during early reperfusion therapy should be further explored in future studies. Given the association among the plasma biomarker, collateral status and clinical outcome 3 month later, FSAP might be of great importance as an adjunct marker for clinical outcome and prognosis in future therapeutic trials targeting collateral circulation, although this would need to be determined in the setting of a prospective trial. Third, continuous intravenous pumping of HMW-HA or those chemically modified HMW-HA with better stability and resistance to enzymatic hydrolysis should be considered in future translational study and clinical practice. Meanwhile, HMW-HA has been reported to play a pivotal role in cytokine release, and glia mediated inflammation.^28,29^ Endothelial FSAP knockout mice should be used in the future study to further elucidate its specific effects. At the molecular level, FSAP might modulate endothelium function by inhibiting Wnt5a signaling and reducing pro-angiogenic factors. The additional molecular information of how FSAP regulates endothelium function could provide detailed mechanism to understand why FSAP levels could predict collateral status, and therapeutic potentials of targeting FSAP.

## Materials and methods

### Ethics approval and consent to participate

The study was approved by Tongji Hospital Research Ethics Committee (IRB ID: TJ-IRB20201022).

### Recruitment of patients

An overview of the study design of the human part is shown in supplementary Fig. 1. Briefly, ischemic stroke patients admitted to the Department of Neurology of Tongji Hospital were enrolled from July 2016 to December 2019. Supplementary Tables 1 and Table 2 provides demographic details of the study participants. In the discovery phase, 40 patients with stroke due to LVO were divided into two groups based on their American Society of Interventional and Therapeutic Neuroradiology/Society of Interventional Radiology (ASTIN/SIR) grades: AIS with good collaterals (ASTIN/SIR 3–4), and AIS with poor collaterals (ASTIN/SIR 0–2). Ten individual samples of equal volume from each group were pooled together for proteomics analysis as previously described.^13^ In the validation phase, another independent population that included 30 healthy controls, 30 patients with AIS due to non-LVO, 30 patients with acute LVO and good collaterals, and 30 patients with acute LVO and poor collaterals were enrolled for Western blots analysis (more details in Supplementary material online, Methods).

### Mouse model of transient focal cerebral ischemia and treatment

All animal studies were approved by the Institute of Animal Care Committee of Tongji Medical College, Huazhong University of Science and Technology, China. All the experimental animals were randomized and all outcome analysis was carried out by independent investigators blinded to the treatment condition. Focal cerebral ischemia was induced by transient Middle Cerebral Artery Occlusion (MCAO) as previously described with modification.^30^ Briefly, a suture was gently advanced to the origin of the middle cerebral artery until regional cerebral blood flow (CBF) was reduced to about 20% of baseline. After 60 min of MCAO, blood flow was restored by removing the suture. After induction of MCAO, the mice were allowed to recover for 1 to 28 days. For FSAP suppression, high-molecular-weight hyaluronan (HMW-HA, 1600 kDa) was purchased from R&D Systems, and was diluted in normal saline at a final concentration of 5 mg/mL and administered intravenously (20 mg/kg) for three consecutive days immediately after transient MCAO^15^ (more details in Supplementary material online, Methods).

### Neurobehavioral tests, immunofluorescence staining, magnetic resonance imaging (MRI) examination, laser speckle imaging, and Western blotting

Detailed descriptions for experimental procedures are provided in the supplementary materials online, Methods.

### Cell culture experiments and treatment

Mouse primary brain microvascular endothelial cells (mBMECs) were isolated from the brain of C57BL/6J mice at P10. Combined oxygen and glucose deprivation (OGD) and reoxygenation were performed as an in vitro model of ischemic stroke as previously described.^31^ Suppression or stimulation of FSAP was induced by HMW-HA or low-molecular-weight hyaluronan (LMW-HA, 20 kDa, R&D Systems) treatment in vitro, respectively. Cells were treated with 10 µg/mL HMW-HA and 500 nM LMW-HA for 1 h ahead of OGD then for another 4 h.^15^ The mBMECs were treated with Wnt5a siRNA for 48 h ahead of OGD, and DKK and rWnt5a were applied during OGD to regulate Wnt5a signaling. The dose and duration of different treatment were selected according to our preliminary experiments and previous studies. (more details in Supplementary material online, Methods)

### Tube formation assay, quantification of apoptotic cells, cell viability assay, transwell migration assay, BrdU cell proliferation assay, trans endothelial electrical resistance (TEER) measurement, and RNA sequencing

Detailed descriptions of experimental procedures are provided in the supplementary materials online, Methods.

### Statistical analysis

Statistical analyses were performed using SPSS 21.0. All data were expressed as the mean ± SD or medians ± interquartile ranges, and significance was determined by two-way analysis of variance (ANOVA) with repeated analysis, Student’s *T*-test, Kruskal–Wallis *H*-test, or one-way ANOVA with Turkey’s post-hoc test to compare differences between groups. All tests were considered statistically significant at *p* < 0.05 (more details in Supplementary material online, Methods).

## Supplementary information

Supplementary Materials

uncut WB images

## Data Availability

The datasets used and/or analyzed during the current study are available from the corresponding authors on reasonable request.
