# Supplementary material for: FSAP aggravated endothelial dysfunction and neurological deficits in acute ischemic stroke due to large vessel occlusion
Source: Signal Transduct Target Ther. 2022 Jan 7;7:6. doi: 10.1038/s41392-021-00802-1 (PMC8738761; doi:10.1038/s41392-021-00802-1)
Supplement: Supplementary file 1 — Supplementary Materials [file 41392_2021_802_MOESM1_ESM.docx]

Supplementary Materials for

FSAP aggravated endothelial dysfunction and neurological deficits in acute ischemic stroke due to large vessel occlusion

Dai-Shi Tian^1,^ ^#^ MD, PhD; Chuan Qin^1, #^ MD, PhD; Luo-Qi Zhou^1, #^ MD, PhD; Sheng Yang^1^ MD, PhD; Man Chen^1^ MD, PhD; Jun Xiao^1^ MD, PhD; Ke Shang^1^ MD, PhD; Dale B. Bosco^2^ PhD; Long-Jun Wu^2^ PhD; Wei Wang^1,^ * MD, PhD

Correspondence to: wwang@tjh.tjmu.edu.cn

**This PDF file includes:**

Materials and Methods

References

Figures. S1 to S7

Tables S1 to S6

Caption for Data S1

**Other Supplementary Materials for this manuscript include the following:**

Data S1 [uncut WB images]

Materials and Methods

**FSAP aggravated endothelial dysfunction and neurological deficits in acute ischemic stroke (AIS) due to large vessel occlusion (LVO)**

**Study design and subjects:**

It is a single-center, retrospective, observational study based on a prospectively collected stroke data base. The clinical diagnosis of acute ischemic stroke was confirmed by cranial magnetic resonance (MR) examinations with diffusion weighted imaging (DWI), and intracranial large vessel status was evaluated by digital subtraction angiography (DSA) within 7 days in every patient. Study-specific inclusion criteria were the presence of intracranial carotid artery terminus or first middle cerebral artery segment (M1) occlusion as confirmed on DSA, and sufficient display of the middle cerebral artery region. Patients with baseline mRS of 0-1 who missed therapeutic time window or refused acute reperfusion therapy were enrolled to rule out the influence from baseline clinical differences and acute reperfusion therapy. Collateral status was assessed using the American Society of Interventional and Therapeutic Neuroradiology/Society of Interventional Radiology (ASTIN/SIR) grading system by blinded observers. Healthy volunteers with no prior history of stroke were matched with patients with AIS due to LVO for age and sex, and were recruited as a healthy control group, to control for the confounding effects of additional risk factors. Cranial MR with DWI and angiography were performed in healthy volunteers to verify that no silent brain infarcts or artery stenosis were present. Patients with AIS in the middle cerebral artery region but no obstructive large vessels were matched with patients with AIS due to LVO for age and sex, and enrolled as AIS due to non-LVO group.

This study was approved by the Ethics Committee of Tongji Medical College, Huazhong University of Science and Technology, Wuhan, China (No. TJ-IRB20201022). All the procedures followed were in accordance with the Helsinki Declaration 2000. Written informed consents were obtained from all patients or their authorizer. Clinical Trial registration: ChiCTR1800015074. Retrospectively registered March 6, 2018. URL of trial registry record: http://www.chictr.org.cn/showproj.aspx?proj=25717

**Sample collection and proteomics analysis**

Blood samples were drawn from subjects within 7 days after stroke onset and the healthy controls, and collected in EDTA Vacutainer tubes (BD, Oxford, UK), centrifuged at 1280 g for 10 minutes at 4 ℃ to separate plasma within 1 hour, and stored at -80 ℃ until required.

For the proteomics analysis, 10 individual samples of equal volume from each group were pooled together, and total proteins were extracted using the cold acetone method. The proteomics analysis, including protein digestion, iTRAQ labelling, strong cation exchange (SCX) fractionation, LC-MS/MS analysis, protein identification, and protein quantitation as described before.^1^

**FSAP ELISA**

A special ELISA to quantify FSAP was established as previously reported ^2,3^ with minor modification, employing immobilized rabbit Anti-FSAP polyclonal antibodies (absin, ab140437) and mouse anti-HABP2 mAb (novusbio, H00003026) for detection. Recombinant human HABP2 protein (MCE, HY-P70832) was used to set up a standard curve. Duoset ancillary reagent kit2 (R&D systems) was used. In brief, after incubation with 10ug/ml rabbit pAb overnigt at 4°C, the 96-well microplate was washed three times with 1X washing buffer. Samples were diluted in reagent diluent concentrate. 100ul diluted standard/samples were applied, and blank/negative controls were also included. After 1h incubation at 37 °C, the plates were washed three times with washing buffer prior to addition of 100ul premixed color reagent to each well. After 30 min incubation at 37 °C, 50ul stop solution was added and the change in OD_450_ was monitored after incubation for 5 min at room temperature. FSAP concentrations were quantified employing the standard curve upon consideration of the sample dilution.

**Statistical analysis**

Statistical analyses were performed using SPSS 21.0 for Windows. The continuous variables were described by means ± SD or by medians and interquartile ranges (IQR), while categorical variables were described by percentages. Mann-Whitney U test, and Kruskal-Wallis H test were used to compare differences between groups.

For prognosis analysis, linear regression analysis was used to investigate the association between plasma FSAP levels and the mRS at 3 months. Patients were then divided into those with good prognosis (mRS 0-2) and those with poor prognosis (mRS 3-6). Adjusted logistic regression model was used to calculate the odds ratio (OR) and 95% confidence intervals (CI), and receiver operating characteristic (ROC) curves were employed to assess the prognostic accuracy of FSAP to poor prognosis. All 60 patients with AIS due to LVO was then further divided into two groups: 28 patients with elevated FSAP level group (1.4 times the median level of FSAP in healthy controls), and the other 32 allocated to normal FSAP level group. All tests were considered statistically significant at p<0.05. Ordinal analysis of mRS was done by FSAP levels allocation under the assumption of proportional odds in the model. The result was expressed as a common OR (OR <1·0 favored elevated FSAP level group) and its 95% CI.

**Pathological effects validation in mouse models of ischemic stroke**

**Animals**

All animal studies were followed the instructions by the Institute of Animal Care Committee of Tongji Medical College, Huazhong University of Science and Technology, China. Generally, adult male C57BL/6 mice (22-27 g; 8-10 weeks old) of SPF grade were obtained from Hunan SJA Laboratory Animal Co. Ltd., Hunan, China. Mice were housed in group as 2-4 mice per cage and kept in a 12-hour light/12-hour dark cycle at 22°C room temperature with water and food ad libitum. The environmental enrichment remained unchanged after surgical procedure and treatment. A total of 179 wild-type mice were used in the experiment. 26 mice died from surgery or post-surgery complications.

All the experimental groups were randomized and all outcome analysis was carried out by independent investigators blinded to the treatment condition. Randomization was performed before surgical procedure by computers using random number generator of GraphPad.

**Mouse model of transient focal cerebral ischemia**

Focal cerebral ischemia was induced by transient Middle Cerebral Artery Occlusion (MCAO) as described before with modyfication.^4^ Briefly, male mice (8-10 weeks, 22-27 g) were anesthetized with isoflurane (3% for induction, 1.5% for maintenance) in mixed O2 and N2O (30%:67%). After a midline skin incision, the common carotid artery was exposed. a monofilament with silicone rubber coated tip of diameter of 0.2 mm (RWD Life Science) was gently advanced from the external carotid artery to the internal carotid artery and further to the origin of the middle cerebral artery (MCA) until regional cerebral blood flow (CBF) was reduced to about 20% of baseline (Figure 3A). After 60 minutes of MCA occlusion, blood flow was restored by removing the suture. After induction of MCAO, the mice were allowed to recover for 1 to 28 d. In sham-operated mice, the same surgical procedure was performed but without occlusion of the middle cerebral artery. Body temperature was measured with a rectal thermometer was maintained at 37.0 ± 0.5°C by a temperature-controlled heating pad during the ischemic period. Regional CBF was measured using a laser speckle contrast imaging (SIM BFI HR, SIM Opto-Technology Co., Ltd, Wuhan, China.) at 15 min before MCAO surgery, 15 min during MCAO period, and 15 min after the onset of reperfusion.

**HMW-HA treatment**

HMW-HA (1600 kDa, R&D Systems) were purchased and dissolved in sterile saline to a concentration of 5 mg/ml for storage under -20 ℃. HMW-HA (20mg/Kg)^5^ or vehicle (equal volume of saline only) was injected through either the lateral tail vein or the retro-orbital venous sinus immediately after MCA occlusion and consecutive daily for 3 days. Suppression of FSAP via HMW-HA treatment was validated as both the expression of brain FSAP and plasma FSAP was greatly reduced at 3d following MCAO. (Figure S4)

**Magnetic Resonance Imaging (MRI) examination**

MRI examination were carried out up to 28 d after MCAO (Figure 2M). Mice were anesthetized to evaluate the infarct volume using a 3.0T MRI scanner (GE Healthcare, Signa HDxt) with an 8-channel head coil by an investigator blinded to the animal group as described before.^6^ The mice’s heads were placed in an animal device with an inner diameter of 30 mm, for signal excitation and detection.

Brain infarct volume was measured on six equally spaced sections encompassing the MCA territory using NIH ImageJ software. These areas were summed and multiplied by the distance between sections (1 mm) to yield a leakage volume in mm3. The actual brain infarct volumes with corrections for edema were calculated as the volume of the contralateral hemisphere minus the non-infarcted volume of the ipsilateral hemisphere. Tissue atrophy was calculated as the volume of the contralateral hemisphere minus the ipsilateral hemisphere.

**Neurobehavioral tests**

Neurobehavioral tests were carried out before and up to 28 d after MCAO (Figure 2B-J). Sensorimotor deficits were evaluated by the foot fault test, rota-rod test, adhesive removal test, and the modified neurological severity scores (mNSS) test. Long-term cognitive deficits were evaluated by the Morris water maze test as described previously.^6-8^

***Foot fault test***. The foot fault test was performed to assess the locomotor function for mice after stroke. Mice were allowed to move on a metal grid surface by gripping the wire with paws and were tested for three trials lasting 2 min each. A foot fault was counted when the forepaw fell or slipped between the wires. Data were expressed as the percentage of error steps to the total moving steps of the contralateral forepaw.

***Rota-rod test***. Mice were placed on a rotating drum (IITC Life Science, Woodland Hills, CA) with speeds accelerating from 5 to 40 rpm within 5 min. The time was recorded when mice fell off the drum (time on rod). Three trials per day were performed for 3 consecutive days before surgery and 4 trials per day were performed 3 d to 28 d after MCAO. The average staying time on rod of three trials 1 d before surgery was used as the pre-surgery baseline value. After surgery, the mean value of trials 2-4 was used as the time on rod of the tested day.

***Adhesive removal test***. A piece of adhesive tape dots (3mm x 3mm) was used to cover the palmar surface of the contralateral forepaw. The time to contact (time to touch) and completely remove the tape (time to remove) from the forepaw was recorded, respectively. Three trials daily were performed for each animal from 3 d before surgery until selected time points after surgery. Mean value of three trials 1 d before surgery was used as the pre-surgery baseline value.

***mNSS test***

The mNSS is a composite of motor, sensory, reflex, and balance tests, grading on a scale ranging from 0 to 18 (normal score, 0; maximal deficit score, 18), including: (1) spontaneous activity, (2) symmetry in limb movement, (3) forepaw outstretching, (4) climbing, (5) body proprioception, and (6) response to vibrissae touch. Higher scores indicated more severe neurological deficit. Neurobehavioral tests were assessed by an investigator blinded to the treatment groups.

***Morris water maze test***. The Morris water maze test was carried out to test 22-27 d after MCAO to evaluate the long-term cognitive functions. Briefly, a circular platform (11 cm ⌀) was submerged in one quadrant of the circular pool (109 cm2) of opaque water. To examine the spatial learning ability, each mouse was placed into the pool from one of the three different start points (3 trials) and allowed to swim for 60 s to locate the hidden platform. The time when the mouse found the hidden platform (Escape latency) was recorded for each trial. When each trial ended, the mouse was allowed to stay on the platform for 20 s to help it remember the external spatial cues displayed around the room. Mice were trained for three consecutive days before, and 22-26 d after MCAO (three trials per day). To evaluate spatial memory, a single 60 s probe trial was performed at 27 d after MCAO when the platform was removed. An investigator recorded the time that each mouse spent in the target quadrant where the platform had previously been placed. Data are expressed as the percentage of the total testing time of 60s (Duration in goal quadrant).

**Laser Speckle Imaging**

A laser speckle imager (SIM BFI HR, SIM Opto-Technology Co., Ltd, Wuhan, China.) was used to monitor the regional cerebral blood flow (CBF) after MCAO as described previously.^9^ Briefly, mice were anesthetized (3% for induction,1.5% for maintenance) and the head was fixed in a head holder in a prone position. A middle incision of scalp was made to expose the skull. The skull surface was illuminated by a laser diode (785 nm) which allowed the laser to penetrate through the brain in a diffused manner. Cerebral blood flow (CBF) was measured by speckle contrast. Two-dimensional microcirculation images were captured 15 min before MCAO, 15 min after the onset of MCAO, and 1d, 3 d, 7 d, 21 d, and 28 d after the onset of reperfusion (**Figure 3A**). For each animal, five consecutive images were captured at each time point. For image data analysis, two identical elliptical regions of interest (ROIs) were created on ipsilateral and the contralateral hemispheres of each image. The blood flow perfusion index was first determined as the ratio of ischemic to non-ischemic CBF and then further normalized to the presurgical baseline to obtain the relative CBF value for each animal.

**Tissue preparation**

At multiple reperfusion time points after MCAO, mice were euthanized with 5% isoflurane and transcardially perfused with 20 ml of 0.9% heparinized PBS. For western blot, brains were removed and rapidly frozen in nitrogen-cooled isopentane. For immunofluorescence, mice were then perfused with 20 ml of 4% paraformaldehyde in 0.1% phosphate buffer. After perfusion brains were removed and postfixed in 4% PFA overnight (4°C) and cryoprotection using 30% sucrose in phosphate-buffered saline (PBS). All brains were stored in -80 °C until processing. Coronal slices of 12 μm were prepared at -20°C using a constant temperature freezing microtome (CM 1900, Leica, Germany). They were then gently transferred to the microslides and stored in -80°C until use.

**FSAP activity assay**

FSAP activity referred to the pro-urokinase, single-chain plasminogen activator urokinase- type (scuPA), activating (pro-fibrinolytic) capacity of FSAP and was determined by a heterogeneous immunocapture activity assay as previously reported ^2,10^. The protease was first bound from a sample to a mAb immobilized (see ELISA) on wells of microplates. After washing the plates, 20mM Sodium citrate solution, 150mM sodium chloride solution, 2%(w/v) BSA, 100mM arginine (pH 6.0) 0.1%(v/v) Tween 80(blocking buffer) were added. The microplates were incubated at 37 °C for 1h, and the blocking solution was discarded. Then, the plasma samples were diluted 1:50 with 20mM Sodium citrate, 150mM sodium chloride solution, 1%(w/v) BSA, 0.1%(v/v) Tween 80, 100 IU/ml heparin. After three wash steps, 100ul each sample were pipetted into the wells. After 1 h incubation at 37 °C, the solution was removed and the plates were washed three times. 50ul single chain urinary-type plasminogen activator (10ug/ml) were added to each well for 2min incubation at RT. Thereafter, 80ul of 1.5mM S2288 in Antithrombin III buffer solution was added to each well. After an incubation for 1h at 37 °C, 50ul of a 50% acetic acid solution was added to stopped the reaction. The change in OD_405_ was monitored. The standard curve was established as described for the ELISA (see earlier). Correspondingly, 1 ml of this recombinant human FSAP protein contains approximately an activity of one plasma equivalent unit per ml (PEU/ml).

**Western blot**

Samples from the 0.9% NaCl perfused mouse cerebral cortex, or primary mBMEC cultures were homogenized in lysis buffer (Beyotime, China) supplemented with phosphatase inhibitors (MedChemExpress Monmouth Junction, NJ). The concentration of protein was measured using bovine serum albumin (BSA, Promoter, China) as a standard. Equal amounts of protein were loaded into 4-15% precast gels and transferred to Nitrocellulose (NC, Satrorius) membranes. Membranes were blocked for 1 h with TBS/0.1%-Tween buffer plus 5% (w/v) non-fat dried milk and incubated overnight at 4°C with primary antibodies diluted in 5% BSA (dissolved in TBS/0.1%-Tween). The primary antibodies used are listed in Table S5; After incubated with DelightTM 800 Conjugate anti-mouse (H+L) and anti-rabbit (H+L) secondary antibody (LI-COR Biosciences), images were captured using Odyssey CLx Imager (LI-COR Biosciences). Secondary antibody controls were performed by incubating the membranes only with secondary antibodies, which yielded no bands. Finally, the intensity of blots was semi-quantified by ImageJ. β-actin was used as the internal control.

The plasma levels of FSAP were also measured by Western blots analysis. Briefly, a portion (20 μg) of each sample extract was then resolved on 12% SDS-PAGE gel electrophoresis, blotted, and incubated overnight with the primary antibodies against FSAP (1:1000, Ab181837, Abcam, UK). Then, membranes were incubated for 1 hour at room temperature with IPKineTM HRP, Goat Anti-Rabbit IgG HCS (Abbkine Scientific) and detected using a ECL Western blotting detection kit (Thermo Scientific). The membranes were stained with Ponceau S as the internal control.

**Immunofluorescence staining**

For immunofluorescence staining, brain slices were acquired through standard procedures as described above. Brain slices were fixed for 15 min in ice-cold 4 % PFA in 0.1 M PBS and then washed and permeablized by 0.25 % Triton-X100 in PBS. After blocking with 10% bovine serum albumin (Sigma–Aldrich) slides were incubated with primary antibodies overnight at 4 ℃ followed by appropriate secondary antibody incubation for 1 h at room temperature. Sections were counterstained with 4,6-diamidino-2-phenylindole (DAPI) (10 µg/mL, Sigma-Aldrich). The primary antibodies used are listed in Table S5.

For brain capillaries quantification, images taken from CD31-stained sections were analyzed with the open source software “AngioTool” (National Cancer Institute, USA) using protocol as previously described^11^ to yield branch length, branch numbers and junction counts. Thresholding was applied to remove small particles so that only actual vessels were quantified.

**Quantitative real-time PCR (qRT-PCR)**

Total RNA was isolated from the normal saline perfused mouse cerebral cortex, or primary mBMEC cultures by using TRIeasy (Yeasen Biotech) according to the manufacturer’s instructions, respectively. ^12^ Quantitative PCR was performed with a Hieff qPCR SYBR Green Master Mix (Yeasen Biotech) using the ABI 7300 real-time PCR system (Applied Biosystems) with target genes and β-actin specific primers. The fold-change in gene expression was calculated using the 2-△△CT method, and β-actin mRNA levels served as an internal reference. Specific primers used for the PCR reaction are presented in detail in Table S6.

**Mechanistical exploration in mouse primary brain microvascular endothelial cells following Oxygen and glucose deprivation**

**Cell cultures**

C57BL/6 mouse primary brain microvascular endothelial cells (mBMECs) were isolated from neonate mice of 10-day-old. Briefly, the brain was first separated and the gray matters were minced into small pieces. Then 25% BSA was used to resuspended the tissue and the homogenate was further centrifugated at 600 x g for 10 min. The 0.1 microvessel pellet located in the lowest layer was transferred and digested with 0.1% collage type II for 35 min at 37 ℃. Finally, the beaded microvessel fragments and individual endothelial cells were obtained in DMEM/F12 medium supplemented with 20% fetal bovine serum (FBS). When the mBMECs reached 90% confluency, the medium was removed. Primary mBMECs were then grown in endothelial culture medium (Sciencell) containing 5 % FBS, 1 % endothelial cell growth supplement and 1 % penicillin/streptomycin solution. All the cells were maintained at 37°C in a humidified atmosphere containing 5% CO2, and cell medium was changed every 2-3 days.

**Oxygen and glucose deprivation (OGD) model**

Combined OGD and reoxygenation were performed as an *in vitro* model of ischemic stroke as previously described.^13^ Briefly, for OGD treatment, mBMECs (90-95% confluency) were washed and incubated with 5% CO2-balanced N2 bubbled glucose-free DMEM, then cells were placed in a hypoxic chamber (model MIC-101, Billups-Rothenberg). The hypoxic environment was achieved by flushing the chamber for 20 min with 5% CO2-balanced N2. The hypoxic chamber was then transferred to a 37°C incubator. Control cells were maintained with normal growth medium in the 37°C incubator under normoxic conditions (5% CO2/95% air). For reoxygenation, the medium of OGD treated cells were replaced with normal growth medium, cells were returned to normoxic conditions in the 37°C incubator.

Primary mBMEC cultures were subjected to 2-8 h OGD and 24 h reoxygenation. Western blot data revealed that expression of FSAP increased with hypoxia, and reached the peak after 4h OGD and 24h reoxygenation. Longer hypoxia did not increase FSAP furtherly. (Figure S3A) The proportions of apoptotic cells in mBMECs were evaluated by flow cytometry-based apoptosis assay utilizing annexin V and PI double-staining. The rate of apoptotic cells increased with the time of hypoxia, while 4h OGD could induce about 30% apoptotic endothelium. (Figure S3B) 4h OGD and 24h reoxygenation was selected for the following experiments.

**Treatment**

**Suppression and stimulation of FSAP**

For suppression assay, high-molecular-weight hyaluronan (HMW-HA, 1600 kDa, R&D Systems) was dissolved in sterile saline and diluted to a concentration gradient (1ug/ml, 10ug/ml, 100ug/ml, 1000ug/ml) to decrease FSAP expression and its enzymatic activity. For stimulation assay, low-molecular-weight HA (LMW-HA, 20 kDa, R&D Systems) was dissolved in sterile saline and diluted to a concentration gradient (10nM, 100nM, 500nM, 1000nM) to increase FSAP expression and its enzymatic activity.^14^

**Inhibition and activation of Wnt5a signaling pathway.**

For inhibition assay, WNT5a gene silencing of mBMECs was performed via WNT5a-siRNA transfection using DharmaFECT™ Transfection Reagents according to the manufacturer’s instructions. 25nM WNT5a siRNA or siNC were transfected to mBMECs 48 h before OGD. Wnt5a inhibitor, recombinant mouse Dickkopf-1 (DKK-1, R&D systems 5897-DK Recombinant Mouse Dkk-1 Protein, 500ng/ml) was applied to mBMECs during OGD.^15^ For activation assay, mBMECs were cultured within 100 ng/mL recombinant mouse WNT5a (rWNT5a, R&D systems, 645-WN Recombinant Human/Mouse Wnt-5a Protein, 200ng/ml) during OGD. ^16^

**Cell viability assay**

5*10^3^ primary mBMECs/well were seeded into 96-well plates. After OGD/R for a designed time or treated with different concentrations of HMW-HA and LMW-HA, cell viability was measured by Cell Counting Kit-8 (CCK8, MedChemExpress). The plates were incubated for 2 h. Afterwards, absorbance at 450 nm was measured to obtain OD value.

**Flow cytometry analysis**

For apoptosis analysis, cells were harvested and stained with annexin V-FITC and propidium iodide (PI; Roche, 1,348,639). The apoptotic cells including both the early apoptotic cells (annexin V-positive and PI-negative) and late apoptotic cells (annexin V-positive and PI-positive) were analyzed using a FACS Calibur flow cytometer (BD Biosciences, USA). mBMEC cultures subjected to 4-8 h OGD exhibited greatly increased apoptosis as shown in Figure S3B.

**BrdU incorporation assay**

For BrdU staining, the cells were incubated with BrdU (Sigma-Aldrich) at a final concentration of 10uM for 4 h prior to fixation. Cells were then treated with 2N HCl at 37℃ for 60 min, followed by treatment with boric acid for 10 min. Anti-BrdU antibody was then added, and the cells were rinsed with PBS, incubated with 488-conjugated secondary antibody and evaluated under a fluorescence microscope.

**Tube formation assay**

Prechilled 96-well plates were coated with 50 μl/well Matrigel (BD Biosciences) and incubated to polymerize at 37˚C for 1 h. Primary mBMECs were re-seeded in Matrigel matrix-coated 96-multiwell plates (2 x 10^4^ cells/well) in endothelial culture medium for 4 h, and plate was carefully washed with culture medium once to remove dead cells and debris. Tubular structures from five views from five wells of each group were photographed using an inverted microscope (Nikon), and quantified by counting the number of branch points and measuring total tube length with ImageJ software. A segment was defined as an element delimited by two junctions of the newly formed tubule network.

**Transwell migration assays**

For cell migration assays, 1 × 10^4^ cells suspended in medium without serum were seeded in the upper chamber (BD Biosciences). Then, 600 μl medium with 10% FBS was added to the lower chamber. After 24 h, the cells on the top surface of the membranes were removed using cotton swabs and the underside of the membrane was fixed in 4% paraformaldehyde for 30 min and stained with 0.1% crystal violet. Then, cells were quantified under a microscope.

**Measurement of BBB permeability *in vitro***

The Trans Endothelial Electrical Resistance (TEER) value and paracellular permeability were measured to reflect the barrier property of the endothelial monolayer *in vitro*. For TEER measurement, mBMECs were seeded on collagen-coated Transwell membranes for 4 days and then exposed to different treatment for 24 hours as described above. Their monolayer electrical resistance was monitored by the CellZscope®-System (NanoaAnalytics GmbH, Muenster, Germany).

After formation of the endothelial monolayer on membrane filters, the paracellular permeability was measured according to the amount of infiltrated fluorescein sodium (375 Da, 5 nl, 1 mg/ml) or FITC-BSA (10 kDa, 5 nl, 0.5 mg/ml) through the endothelial monolayer.

**RNA Sequencing of mBMECs *in vitro***

Total RNA was extracted using Trizol reagent kit (Invitrogen, Carlsbad, CA,USA) according to the manufacturer’s protocol. The mRNA was enriched, fragmented and reverse transcripted into cDNA. Then the cDNA fragments were purified with QiaQuick PCR extraction kit (Qiagen, Venlo, The Netherlands), end repaired, PCR amplified, and sequenced using Illumina Novaseq6000.

Reads obtained were further filtered by fastp (version 0.18.0). Ribosome RNA was removed and paired-end clean reads were mapped to the reference genome using HISAT2. 2.4 and assembled by using StringTie v1.3.1. For each transcription region, a FPKM (fragment per kilobase of transcript per million mapped reads) value was calculated to quantify its expression abundance and variations, using RSEM software. Differential expression genes (DEGs) analysis was performed by DESeq2 software. All DEGs were mapped to GO terms in the Gene Ontology database (<http://www.geneontology.org/>), and pathway enrichment analysis was performed using KEGG database. All downstream statistical analyses and generating plots were performed in R environment (v3.6.3) (http://www.r-project.org/). An exploratory P value <0.05 was used to determine statistical significance.

**References**

1 Qin, C. et al. Proteomic profiling of plasma biomarkers in acute ischemic stroke due to large vessel occlusion. J Transl Med. 17, 214, (2019).

2 Romisch, J., Feussner, A. & Stohr, H. A. Quantitation of the factor VII- and single-chain plasminogen activator-activating protease in plasmas of healthy subjects. Blood Coagul Fibrinolysis. 12, 375-383, (2001).

3 Hanson, E. et al. Plasma factor VII-activating protease antigen levels and activity are increased in ischemic stroke. J Thromb Haemost. 10, 848-856, (2012).

4 Xie, M. et al. Glial gap junctional communication involvement in hippocampal damage after middle cerebral artery occlusion. Ann Neurol. 70, 121-132, (2011).

5 Mattheolabakis, G., Milane, L., Singh, A. & Amiji, M. M. Hyaluronic acid targeting of CD44 for cancer therapy: from receptor biology to nanomedicine. J Drug Target. 23, 605-618, (2015).

6 Li, X. et al. Fingolimod suppresses neuronal autophagy through the mTOR/p70S6K pathway and alleviates ischemic brain damage in mice. PloS one. 12, e0188748, (2017).

7 Chen, M. et al. Deficiency of microglial Hv1 channel is associated with activation of autophagic pathway and ROS production in LPC-induced demyelination mouse model. J Neuroinflammation. 17, 333, (2020).

8 Sun, P. et al. Endothelium-Targeted Deletion of microRNA-15a/16-1 Promotes Poststroke Angiogenesis and Improves Long-Term Neurological Recovery. Circulation Research. 126, 1040-1057, (2020).

9 Liu, X. et al. Polyunsaturated fatty acid supplement alleviates depression-incident cognitive dysfunction by protecting the cerebrovascular and glymphatic systems. Brain Behav Immun. 89, 357-370, (2020).

10 Stephan, S. et al. Direct chromogenic substrate immuno-capture activity assay for testing of factor VII-activating protease. Clin Chem Lab Med. 49, 1199-1204, (2011).

11 Zudaire, E., Gambardella, L., Kurcz, C. & Vermeren, S. A computational tool for quantitative analysis of vascular networks. PLoS One. 6, e27385, (2011).

12 Qin, C. et al. Fingolimod Protects Against Ischemic White Matter Damage by Modulating Microglia Toward M2 Polarization via STAT3 Pathway. Stroke. 48, 3336-3346, (2017).

13 Li, C. Y. et al. Inhibition of mTOR pathway restrains astrocyte proliferation, migration and production of inflammatory mediators after oxygen-glucose deprivation and reoxygenation. Neurochem Int. 83-84, 9-18, (2015).

14 Mambetsariev, N. et al. Hyaluronic Acid binding protein 2 is a novel regulator of vascular integrity. Arterioscler Thromb Vasc Biol. 30, 483-490, (2010).

15 Dong, Z. et al. Sulphonated Formononetin Induces Angiogenesis through Vascular Endothelial Growth Factor/cAMP Response Element-Binding Protein/Early Growth Response 3/Vascular Cell Adhesion Molecule 1 and Wnt/beta-Catenin Signaling Pathway. Pharmacology. 101, 76-85, (2018).

16 Ekstrom, E. J. et al. WNT5A induces release of exosomes containing pro-angiogenic and immunosuppressive factors from malignant melanoma cells. Mol Cancer. 13, 88, (2014).


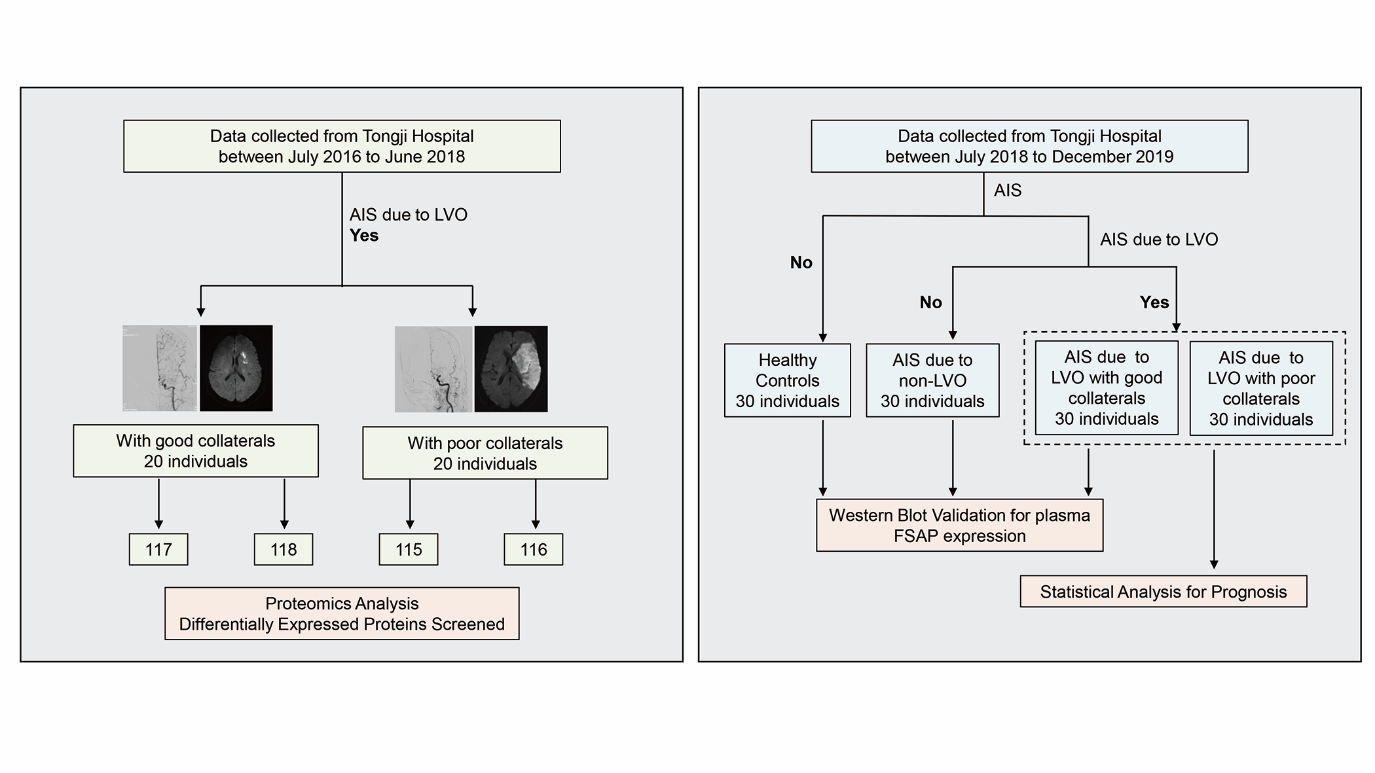
Figure. S1.

Flowchart of the procedures in the evaluation of differentially expressed proteins and validation for FSAP expression in patients with AIS due to LVO but different collaterals.


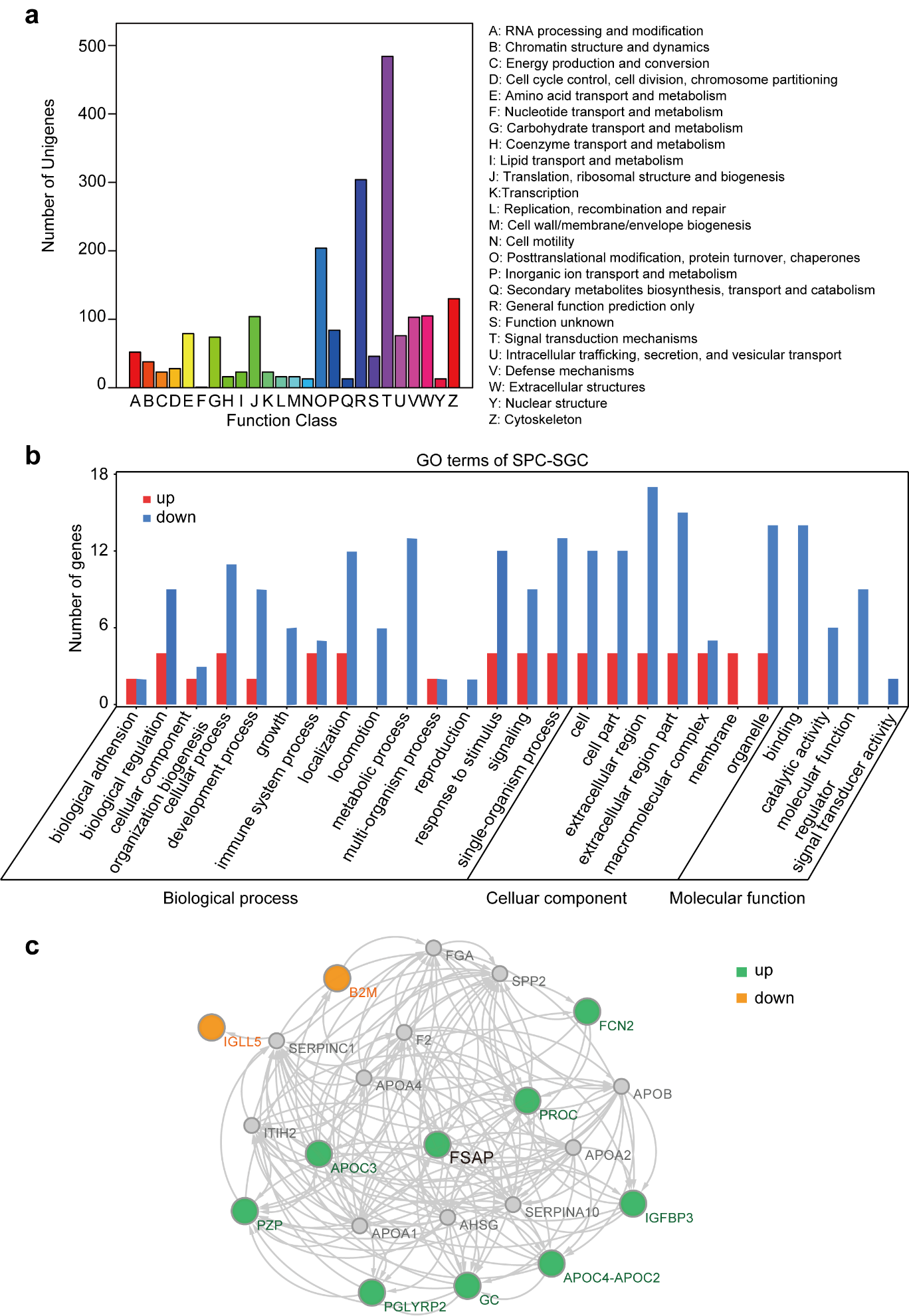


**Figure. S2.**

Categorization and functional annotation of differentially expressed proteins with AIS due to LVO but different collaterals based on proteomic analysis.

a. Histogram presentation of KOG classification.

b. Categorization and functional annotation of differentially expressed transcripts based on gene ontology, including biological process, cellular component and molecular function.

c. Protein-protein interaction (PPI) network of the 11 differentially expressed proteins. PPI information of the proteins was acquired from the STRING database; the PPI networks was constructed using Cytoscape3.8.2 software.


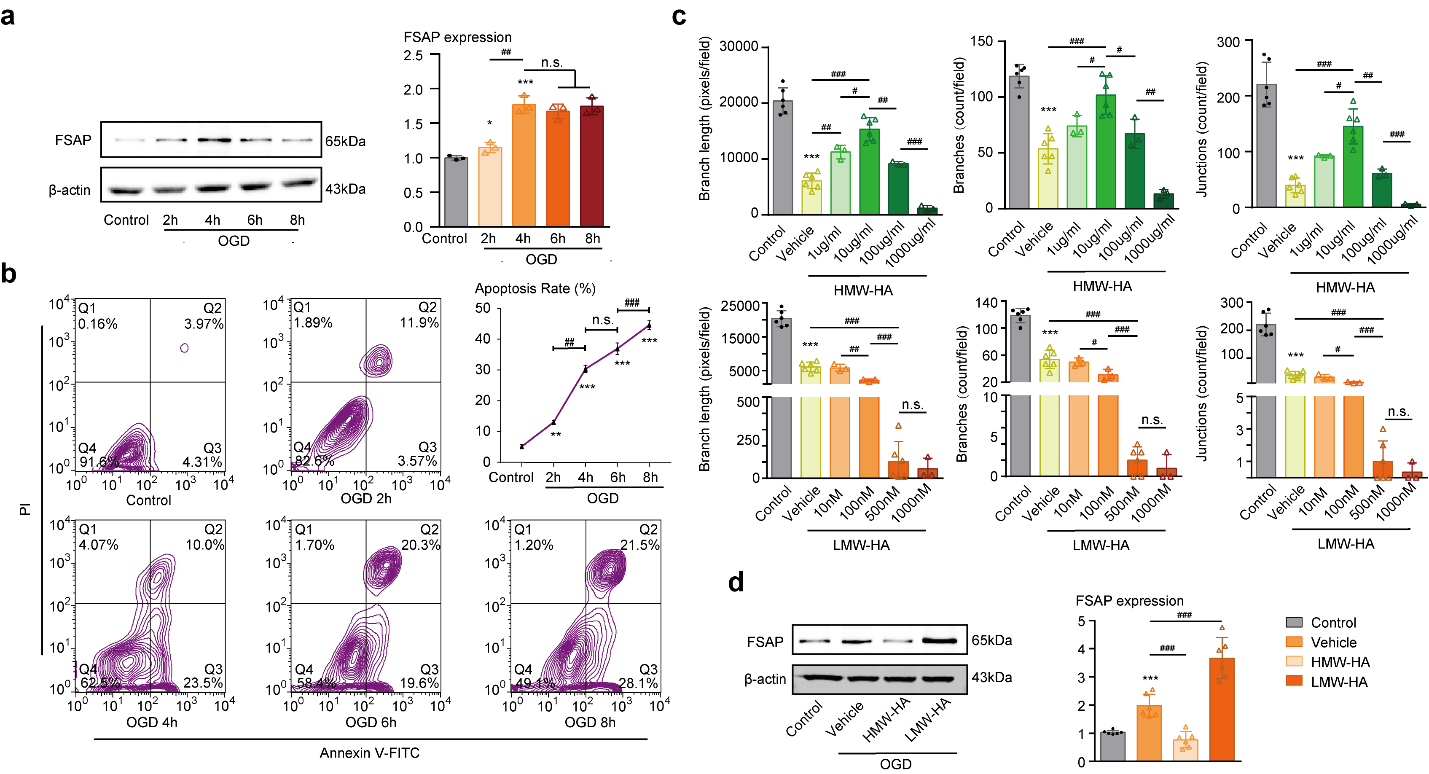


Figure. S3.

Suppression or stimulation of FSAP enhances or suppresses the tube formation in mBMECs cultures following OGD in a dose-dependent manner, respectively.

a. Endothelial FSAP expression following OGD was detected by Western blotting. Representative Western blot images and quantitative analysis. Data is expressed as mean ± SD; n = 3 for each group. *p < 0.05, ***p < 0.001 versus Control group, n.s. not significant, ##p < 0.01, horizontal lines and corresponding hashes compare samples aligned with each end of the horizontal line by one-way ANOVA followed by Tukey’s multiple comparison tests.

b. Apoptotic cells were stained with annexin V-FITC and propidium iodide and analyzed using a FACS Calibur flow cytometer. Representative images of FACS analysis of apoptotic cells and quantitative analysis of apoptotic rates. Data is expressed as mean ± SD; n =3 for each group; **p < 0.01, ***p < 0.001 versus Control group, n.s. not significant, #p < 0.05, ##p < 0.01 versus Vehicle group by one-way ANOVA followed by Tukey’s multiple comparison tests.

c. Suppression or stimulation of FSAP’s effect on mBMECs via different dose of HMW-HA and LMW-HA was evaluated by tube formation assays. Quantitative analysis of branch length, branch counts, and junction counts. Data is expressed as mean ± SD; n = 3-6 for each group; ***p < 0.001 versus Control group, n.s. not significant, #p < 0.05, ##p < 0.01, ###p < 0.001, horizontal lines and corresponding hashes compare samples aligned with each end of the horizontal line by one-way ANOVA followed by Tukey’s multiple comparison tests.

d. Suppression and stimulation of FSAP via 10ug/ml HMW-HA and 500nM LMW-HA were evaluated by Western blotting. Representative Western blot images and quantitative analysis. Data is expressed as mean ± SD; n = 6 for each group; ***p < 0.001 versus Control group, ###p < 0.001 versus Vehicle group by one-way ANOVA followed by Tukey’s multiple comparison tests.

­­
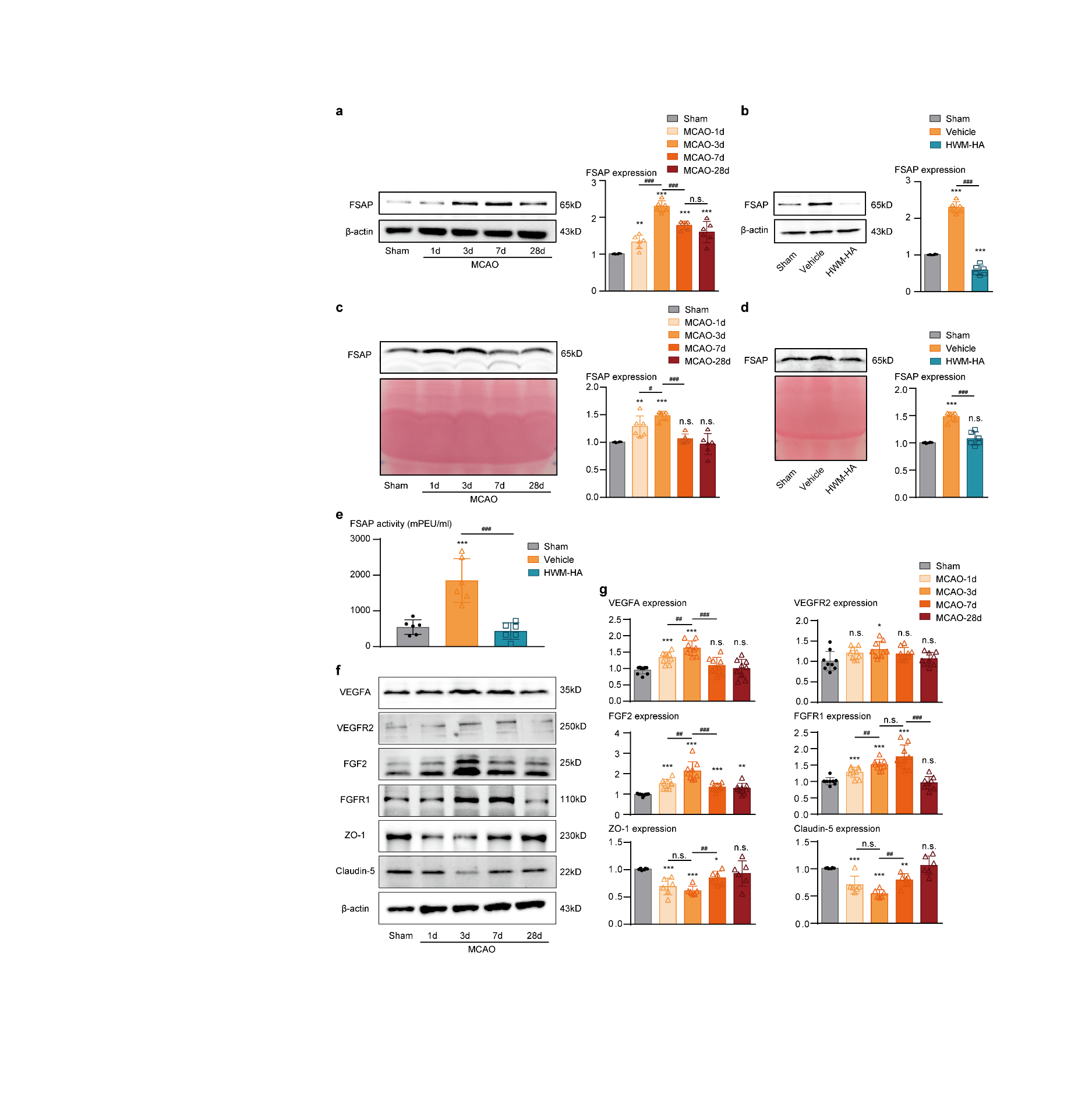


Figure. S4.

Pathological expression of FSAP and vascular factors in mouse model of ischemic stroke

a-b. Brain and plasma FSAP levels in mouse model of ischemic stroke were evaluated by Western blotting. Representative Western blot images of (a) brain FSAP and (b) plasma FSAP, and quantitative analysis. Data is expressed as mean ± SD; n = 6 for each group; n.s. not significant, **p < 0.01, ***p < 0.001 versus Sham group, n.s. not significant, #p < 0.05, ###p < 0.001, horizontal lines and corresponding hashes compare samples aligned with each end of the horizontal line by one-way ANOVA followed by Tukey’s multiple comparison tests.

c-d. Brain and plasma FSAP levels following MCAO and HMW-HA treatment were evaluated by Western blotting. Representative western blot images of (c) brain FSAP and (d) plasma FSAP and quantitative analysis. Data is expressed as mean ± SD; n = 6 for each group; n.s. not significant, ***p < 0.001 versus Sham group, n.s. not significant, ###p < 0.001 versus Vehicle group by one-way ANOVA followed by Tukey’s multiple comparison tests.

e. Plasma FSAP activity following MCAO and HMW-HA treatment was evaluated by a heterogeneous immunocapture activity assay. Data is expressed as mean ± SD; n = 6 for each group; ***p < 0.001 versus Sham group, ###p < 0.001 versus Vehicle group by one-way ANOVA followed by Tukey’s multiple comparison tests.

f-g. Proangiogenic factors and blood-brain barrier markers were evaluated by Western blot. (f) Representative western blot images of proangiogenic factors (VEGFA, VEGFR2, FGF2, and FGFR1) and blood-brain barrier markers (ZO-1, and Claudin-5), and (g) quantitative analysis. Data is expressed as mean ± SD; n = 6-9 for each group; n.s. not significant, *p < 0.05, **p < 0.01, ***p < 0.001 versus Sham group, n.s. not significant, ##p < 0.01, ###p < 0.001, horizontal lines and corresponding hashes compare samples aligned with each end of the horizontal line by one-way ANOVA followed by Tukey’s multiple comparison tests.


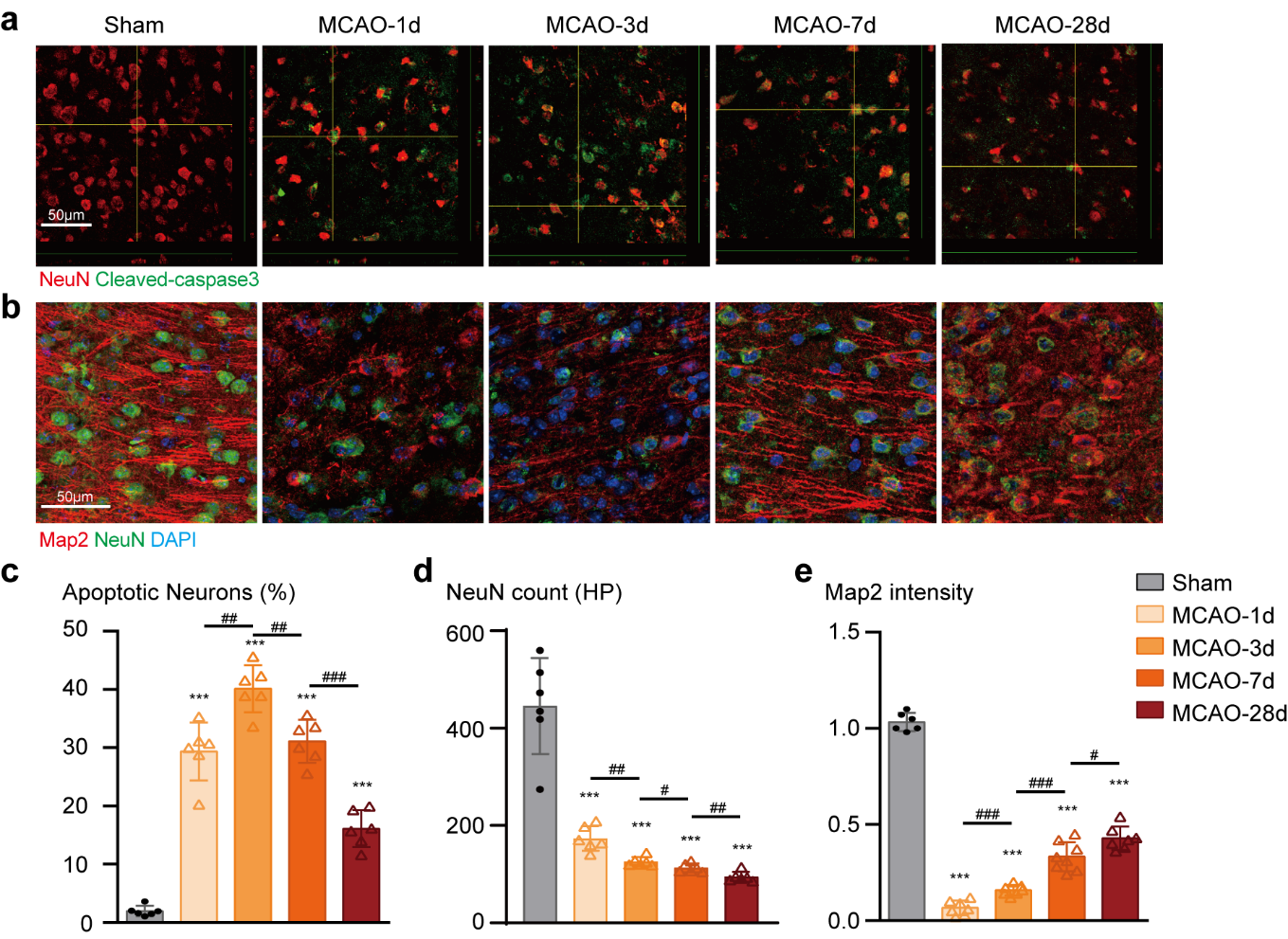


Figure. S5.

Neuron apoptosis and loss following MCAO

a-e, Neuron apoptosis and loss were measured and quantified by immunostaining. (a) Representative cleaved-caspase 3 (green) and NeuN (red) immunofluorescent images, (b) representative NeuN (green) and Map2 (red) immunofluorescent images, and quantitative analysis (c, apoptotic neurons, d, Neuron count, e, Map2 intensity). Data is expressed as mean ± SD; n = 6-8 for each group; ***p < 0.001, horizontal lines and corresponding asterisks compare samples aligned with each end of the horizontal line by one-way ANOVA followed by Tukey’s multiple comparison tests.


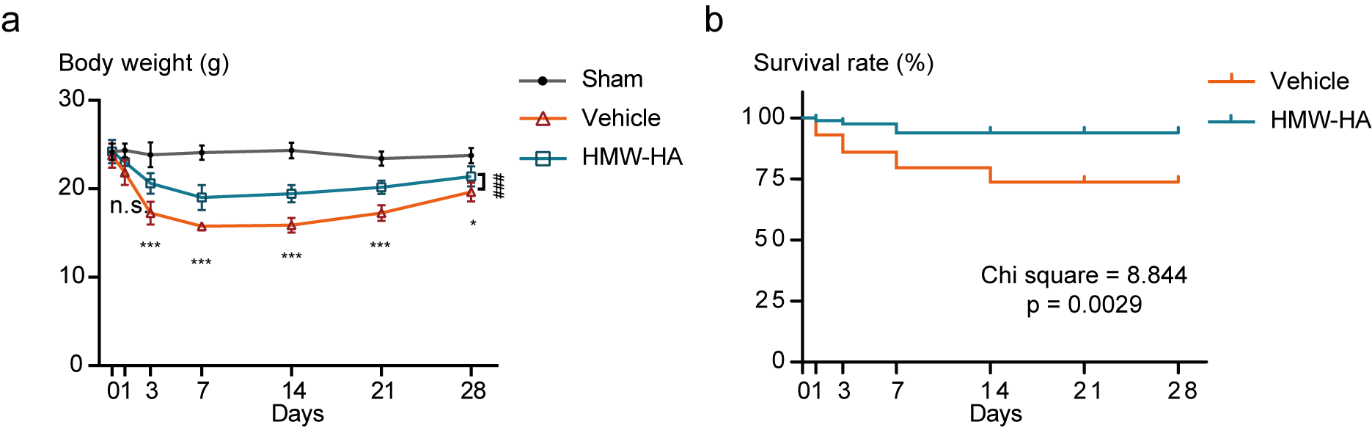


Figure. S6.

Changes of body weight and survival curve following MCAO and HMW-HA treatment.

a, Sequential tracing of body weight in mice. Data is represented as mean ± SD; n = 8-12 for each group.

b, The survival curve of HMW-HA and Vehicle mice after MCAO was traced and each death event was recorded immediately. The overall survival rate was significantly better for mice with HMW-HA administration (Chi square = 8.844, p = 0.0029).


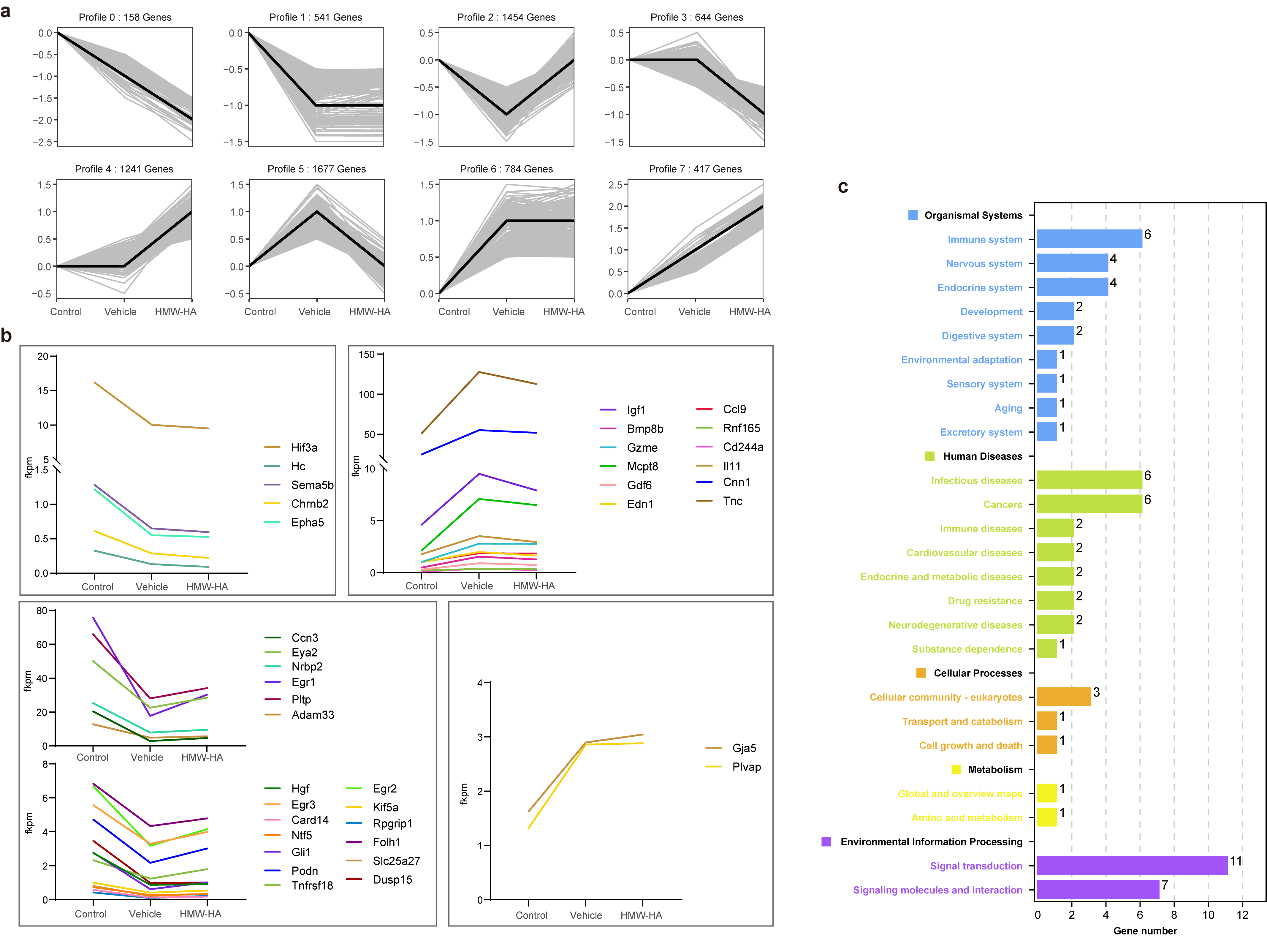
>

Figure. S7.

Identification of the transcriptional changes of the 38 selected genes.

a, Trend analysis of gene expression profiles. The numbers in the lower left corner indicate p-values in the profile compared to other profiles. Eight clusters were identified. For each cluster, average expression at each group is plotted in black. The HMW-HA affected trend was observed in cluster 2, 4 and 5.

b, The 38 selected genes were broken down into four different groups. Group 1 (left top) down-regulated in Vehicle group and more decreased after HMW-HA treatment. Group 2 (left bottom) reached lowest expression in Vehicle group and upregulated after HMW-HA treatment. Group 3 (right top) peaked in the Vehicle group, and down-regulated after HMW-HA treatment, Group 4 (right bottom) consisted of genes that up-regulated in the Vehicle group more increased after HMW-HA treatment.

c, KEGG pathways of 38 selected genes were identified

Table S1.

Baseline characteristics of AIS patients due to LVO with different collateral circulation in iTRAQ-based quantitative proteomics analysis

|  |  |  |  |  |  |  |  |
| --- | --- | --- | --- | --- | --- | --- | --- |
|  | **Demographic and clinical characteristics of the study groups** | |  | **AIS patients due to LVO** | | |  |
|  |  |  |  | **with good collaterals (n=20)** | **with poor collaterals (n=20)** | **P value** |  |
|  | **Characteristics** | |  |  |  |  |  |
|  |  | Age, median (IQR), y |  | 60 (52-63.3) | 52.5 (44-63) | 0.128 |  |
|  |  | Female, n (%) |  | 6 (30.0) | 9 (45.0) | 0.340 |  |
|  |  | NIHSS score at baseline |  | 5.7 (3.0) | 7.0 (3.1) | 0.217 |  |
|  | **Medical history, n (%)** | |  |  |  |  |  |
|  |  | Hypertension |  | 15 (75.0) | 12 (60.0) | 0.324 |  |
|  |  | Diabetes mellitus |  | 7 (35.0) | 3 (15.0) | 0.152 |  |
|  |  | coronary heart disease |  | 3 (15.0) | 0 (0.0) | 0.075 |  |
|  |  | Hypercholesterolemia |  | 4 (20.0) | 1 (5.0) | 0.159 |  |
|  |  | Current or previous smoking |  | 8 (40.0) | 10 (50.0) | 0.537 |  |
|  |  | Heavy alcohol use |  | 6 (30.0) | 4 (20.0) | 0.478 |  |
|  | **Blood Test, mean (SD)** | |  |  |  |  |  |
|  |  | HCY |  | 16.8 (9.3) | 15.3 (8.4) | 0.621 |  |
|  |  | cholesterol |  | 3.7 (1.0) | 3.7 (0.9) | 0.993 |  |
|  |  | triglyceride |  | 1.3 (0.7) | 1.5 (0.7) | 0.446 |  |
|  |  | LDL |  | 2.2 (0.8) | 2.1 (0.8) | 0.930 |  |
|  |  | CRP |  | 3.2 (5.4) | 4.9 (7.8) | 0.429 |  |

Data are shown as mean (SD), median (IQR) for continuous variables, and as percentages for categorical variables.

AIS, acute ischemic stroke; IQR, interquartile range; HCY, homocysteine; LDL, low-density lipoprotein; CRP, C-reactive protein.

Table S2.

Baseline characteristics of patients and controls in Western blot validation

|  |  |  |  |  |  |  |  |  |  |  |  |  |  |
| --- | --- | --- | --- | --- | --- | --- | --- | --- | --- | --- | --- | --- | --- |
|  | **Demographic and clinical characteristics of the study groups** | |  | **Healthy Controls (n=30)** | **AIS patients due to non-LVO (n=30)** | **AIS patients due to LVO (n=60)** | **P^a^ value** | **P^b^ value** | **P^c^ value** | **AIS patients due to LVO** | | **P^d^ value** |  |
|  |  |  |  |  |  |  |  |  |  | **with good collaterals (n=30)** | **with poor collaterals (n=30)** |  |  |
|  | **Characteristics** | |  |  |  |  |  |  |  |  |  |  |  |
|  |  | Age, median (IQR), y |  | 54.5 (43.25-65.75) | 55.5 (48.25-64.5) | 54 (46.75-63) | 0.899 | 0.903 | 0.974 | 55 (49.5-62.75) | 54 (44-63) | 0.724 |  |
|  |  | Female, n (%) |  | 11 (36.7) | 8 (27.0) | 18 (30.0) | 0.414 | 0.529 | 0.746 | 8 (27.0) | 10 (33.0) | 0.581 |  |
|  |  | NIHSS score at baseline |  | NA | 5.9 (4.3) | 6.6 (3.8) | NA | NA | 0.439 | 6.1 (4.2) | 7.1(3.3) | 0.350 |  |
|  | **Medical history, n (%)** | |  |  |  |  |  |  |  |  |  |  |  |
|  |  | Hypertension |  | 5 (16.7) | 17 (57.0) | 42 (70.0) | 0.001 | 0.000 | 0.214 | 20 (66.7) | 22 (73.3) | 0.581 |  |
|  |  | Diabetes mellitus |  | 1 (3.3) | 7 (23.0) | 15 (25.0) | 0.023 | 0.011 | 0.864 | 8 (26.7) | 7 (23.3) | 0.770 |  |
|  |  | coronary heart disease |  | 2 (6.7) | 1 (3.3) | 7 (12.0) | 0.561 | 0.462 | 0.194 | 5 (16.7) | 2 (6.7) | 0.235 |  |
|  |  | Hypercholesterolemia |  | 3 (10.0) | 3 (9.1) | 9 (15.0) | 0.694 | 0.516 | 0.834 | 4 (13.3) | 5 (16.7) | 0.723 |  |
|  |  | Current or previous smoking |  | 2 (6.7) | 4 (13.0) | 28 (47.0) | 0.000 | 0.000 | 0.238 | 15 (50.0) | 13 (43.3) | 0.612 |  |
|  |  | Heavy alcohol use |  | 5 (16.7) | 9 (30.0) | 18 (30.0) | 0.229 | 0.175 | 1.000 | 11 (36.7) | 7 (23.3) | 0.267 |  |
|  | **Blood Test, mean (SD)** | |  |  |  |  |  |  |  |  |  |  |  |
|  |  | HCY |  | 10.9 (2.9) | 13.4 (5.3) | 13.9 (7.6) | 0.029 | 0.044 | 0.759 | 14.3 (9.3) | 13.6 (5.5) | 0.723 |  |
|  |  | cholesterol |  | 3.9 (0.8) | 3.9 (1.1) | 3.7 (1.1) | 0.962 | 0.314 | 0.376 | 3.8 (1.1) | 3.7 (1.1) | 0.784 |  |
|  |  | triglyceride |  | 1.2 (0.6) | 1.4 (0.6) | 1.5 (1.0) | 0.313 | 0.184 | 0.581 | 1.4 (0.7) | 1.6 (1.3) | 0.419 |  |
|  |  | LDL |  | 2.4 (0.7) | 2.5 (1.0) | 2.3 (0.9) | 0.645 | 0.653 | 0.374 | 2.3 (0.9) | 2.3 (0.9) | 0.954 |  |
|  |  | CRP |  | 4.7 (12.4) | 5.5 (7.1) | 9.4 (22.7) | 0.773 | 0.297 | 0.360 | 5.1 (7.4) | 13.8 (30.7) | 0.142 |  |
|  |  |  |  |  |  |  |  |  |  |  |  |  |  |

Data are shown as mean (SD), median (IQR) for continuous variables, and as percentages for categorical variables.

Table S3.

List of differentially expressed protein between SGC and SPC group.

| Gene Symbol | Description | SPC | SGC | Fold change (SGC/SPC) | P-value |  |
| --- | --- | --- | --- | --- | --- | --- |
| PZP | Pregnancy-zone protein | 1.136 | 0.658 | 0.628521 | 0.0350 |  |
| HABP2 | Hyaluronan binding protein 2 | 1.0255 | 0.747 | 0.78425 | 0.0299 |  |
| APOC3 | Apolipoprotein C-III | 0.785 | 0.5805 | 0.73949 | 0.0404 |  |
| GC | Group-specific component | 1.047 | 1.0345 | 0.763106 | 0.0236 |  |
| APOC4-APOC2 | APOC4-APOC2 readthrough | 0.6545 | 0.5265 | 0.804431 | 0.0466 |  |
| PGLYRP2 | Peptidogylcan recognition protein 2 | 1.027 | 0.8385 | 0.816456 | 0.0070 |  |
| PROC | Protein C | 0.8905 | 0.965 | 0.818496 | 0.0288 |  |
| FCN2 | Ficolin2 | 1.1325 | 0.9315 | 0.822517 | 0.0338 |  |
| IGFBP3 | Insulin like growth factor binding protein 3 | 0.994 | 0.824 | 0.828974 | 0.0207 |  |
| IGLL5 | Immunoglobulin lambda-like polypeptide 5 | 0.919 | 1.304 | 1.418934 | 0.0431 |  |
| B2M | Beta-2-microglobulin | 0.830 | 1.2875 | 1.551205 | 0.0434 |  |

AIS, acute ischemic stroke; IQR, interquartile range; HCY, homocysteine; LDL, low-density lipoprotein; CRP, C-reactive protein .

a Patients with AIS due to non-LVO versus Healthy Controls.

b Patients with AIS due to LVO versus Healthy Controls.

c Patients with AIS due to LVO versus patients with AIS due to non-LVO.

d AIS Patients due to LVO with good collaterals versus patients with poor collaterals.

Table S4.

List of the 38 differentially expressed genes selected based on GO term analysis and through existing literature describing endothelial function.

| **Gene Symbol** | **Description** | **Control FPKM** | **Vehicle FPKM** | **HMW-HA**  **FPKM** |  |
| --- | --- | --- | --- | --- | --- |
| **Cnn1** | calponin 1 | 24.72 | 55.08 | 51.65 |  |
| **Folh1** | folate hydrolase 1 | 6.83 | 4.34 | 4.79 |  |
| **Bmp8b** | bone morphogenetic protein 8b | 0.49 | 1.54 | 1.27 |  |
| **Hif3a** | hypoxia inducible factor 3, alpha subunit | 16.19 | 10.06 | 9.52 |  |
| **Il11** | interleukin 11 | 1.77 | 3.52 | 2.94 |  |
| **Cd244a** | CD244 molecule A | 0.11 | 0.37 | 0.25 |  |
| **Card14** | caspase recruitment domain family, member 14 | 0.58 | 0.12 | 0.18 |  |
| **Pltp** | phospholipid transfer protein | 65.85 | 28.03 | 34.09 |  |
| **Eya2** | EYA transcriptional coactivator and phosphatase 2 | 49.99 | 22.49 | 28.48 |  |
| **Ccl9** | chemokine (C-C motif) ligand 9 | 1.04 | 1.86 | 1.84 |  |
| **Igf1** | insulin-like growth factor 1 | 4.61 | 9.49 | 7.90 |  |
| **Edn1** | endothelin 1 | 1.00 | 1.99 | 1.64 |  |
| **Gzme** | granzyme E | 1.02 | 2.79 | 2.76 |  |
| **Mcpt8** | mast cell protease 8 | 2.13 | 7.08 | 6.50 |  |
| **Slc25a27** | solute carrier family 25, member 27 | 0.8 | 0.22 | 0.34 |  |
| **Gli1** | GLI-Kruppel family member GLI1 | 2.76 | 0.61 | 1.02 |  |
| **Rnf165** | ring finger protein 165 | 0.22 | 0.36 | 0.35 |  |
| **Hc** | hemolytic complement | 0.33 | 0.14 | 0.09 |  |
| **Adam33** | a disintegrin and metallopeptidase domain 33 | 12.77 | 4.81 | 5.64 |  |
| **Chrnb2** | cholinergic receptor, nicotinic, beta polypeptide 2 (neuronal) | 0.61 | 0.29 | 0.22 |  |
| **Tnc** | tenascin C | 50.98 | 127.65 | 112.61 |  |
| **Podn** | podocan | 4.72 | 2.17 | 3.02 |  |
| **Hgf** | hepatocyte growth factor | 2.75 | 0.87 | 0.91 |  |
| **Epha5** | Eph receptor A5 | 1.22 | 0.55 | 0.53 |  |
| **Egr3** | early growth response 3 | 5.57 | 3.29 | 3.99 |  |
| **Plvap** | plasmalemma vesicle associated protein | 1.32 | 2.86 | 2.88 |  |
| **Ccn3** | cellular communication network factor 3 | 20.36 | 2.85 | 4.61 |  |
| **Egr2** | early growth response 2 | 6.68 | 3.18 | 4.15 |  |
| **Egr1** | early growth response 1 | 75.72 | 17.68 | 30.24 |  |
| **Tnfrsf18** | tumor necrosis factor receptor superfamily, member 18 | 2.32 | 1.24 | 1.80 |  |
| **Dusp15** | dual specificity phosphatase-like 15 | 3.46 | 0.98 | 1.01 |  |
| **Gdf6** | growth differentiation factor 6 | 0.29 | 0.90 | 0.74 |  |
| **Sema5b** | sema domain, seven thrombospondin repeats (type 1 and type 1-like), transmembrane domain (TM) and short cytoplasmic domain, (semaphorin) 5B | 1.28 | 0.65 | 0.60 |  |
| **Gja5** | gap junction protein, alpha 5 | 1.63 | 2.90 | 3.04 |  |
| **Rpgrip1** | retinitis pigmentosa GTPase regulator interacting protein 1 | 0.42 | 0.093 | 0.22 |  |
| **Ntf5** | neurotrophin 5 | 0.70 | 0.27 | 0.33 |  |
| **Kif5a** | kinesin family member 5A | 1.00 | 0.41 | 0.54 |  |
| **Nrbp2** | nuclear receptor binding protein 2 | 25.23 | 7.89 | 9.54 |  |
|  |  |  |  |  |  |

Table S5.

List of the primary antibodies used in the present study.

| **Target antigen** | **Vendor or Source** | **Catalog #** | **Working concentration** | **RRID**  **(preferred but not required)** | **Persistent ID / URL** |
| --- | --- | --- | --- | --- | --- |
| Mouse  CD31 | BD Pharmingen | 553370 | 1:50 |  | https://www.bdbiosciences.com/cn/applications/research/stem-cell-research/cancer-research/mouse/purified-rat-anti-mouse-cd31-mec-133/p/553370 |
| Mouse  FSAP | Abcam | Ab181837 | 1:2000 |  | https://www.abcam.cn/habp2-antibody-epr14551-ab181837.html |
| BrdU | Abcam | Ab6236 | 1:200 |  | https://www.abcam.cn/brdu-antibody-bu175-icr1-proliferation-marker-ab6326.html |
| Mouse  Cleaved caspases-3 | Cell signaling | 9661 |  |  | https://www.cellsignal.cn/products/primary-antibodies/cleaved-caspase-3-asp175-antibody/9661 |
| Mouse  Map2 | Cell signaling | 4542 | 1:200 |  | https://www.cellsignal.cn/products/primary-antibodies/map2-antibody/4542 |
| Mouse  NeuN | Millipore | MAB377 |  |  | https://www.merckmillipore.com/CN/zh/product/Anti-NeuN-Antibody-clone-A60,MM_NF-MAB377 |
| Mouse  VEGFA | ABclonal Technology | A12303 | 1:1000 | [AB_2759160](http://antibodyregistry.org/AB_2759160) | https://abclonal.com.cn/catalog/A12303 |
| Mouse  VEGFR2 | Proteintech | 26415-1-AP | 1:1000 |  | https://www.ptgcn.com/products/VEGFR2-Antibody-26415-1-AP.html |
| Mouse  FGF2 | ABclonal Technology | A0235 | 1:1000 | [AB_2757048](http://antibodyregistry.org/AB_2757048) | https://abclonal.com.cn/catalog/A0235 |
| Mouse  FGFR1 | ABclonal Technology | A0082 | 1:1000 | [AB_2756937](http://antibodyregistry.org/AB_2756937) | https://abclonal.com.cn/catalog/A0082 |
| Mouse  Wnt5a | Proteintech | 55184-1-AP | 1:1000 |  | https://www.ptgcn.com/products/WNT5A-B-Antibody-55184-1-AP.html |
| Mouse  β-catenin | Proteintech | 17565-1-AP | 1:1000 |  | https://www.ptgcn.com/products/b-cat-Antibody-17565-1-AP.html |
| Mouse  ZO-1 | [Thermo Fisher Scientific](https://www.thermofisher.com/cn/zh/home/brands/invitrogen.html) | 61-7300 | 1:1000 | AB_2533938 | https://www.thermofisher.com/cn/zh/antibody/product/ZO-1-Antibody-Polyclonal/61-7300 |
| Mouse  Claudin 5 | [Thermo Fisher Scientific](https://www.thermofisher.com/cn/zh/home/brands/invitrogen.html) | 35-2500 | 1:1000 | AB_2533200 | https://www.thermofisher.com/cn/zh/antibody/product/Claudin-5-Antibody-clone-4C3C2-Monoclonal/35-2500 |
| Mouse  occludin | [Thermo Fisher Scientific](https://www.thermofisher.com/cn/zh/home/brands/invitrogen.html) | 71-1500 | 1:1000 | AB_2533977 | https://www.thermofisher.com/cn/zh/antibody/product/Occludin-Antibody-Polyclonal/71-1500 |
| Mouse  albumin | Proteintech | 16475-1-AP | 1:200 |  | http://www.ptgcn.com/products/ALB-Antibody-16475-1-AP.html |

Table S6.

List of specific primers used for the PCR reaction.

| **Gene** | **Forward Primer** | **Reverse Primer** |
| --- | --- | --- |
| β-Actin | TGGAATCCTGTGGCATCCATGA | AATGCCTGGGTACATGGTGGTA |
| HABP2 | CATTGGGCTCTCACTGATGTC | CTGAAGGTATCCCCTCTGATGAT |
| Hif3a | GAAGTTCACATACTGCGACGA | GTCCAAAGCGTGGATGTATTCAT |
| Hc | GAACAAACCTACGTCATTTCAGC | GTCAACAGTGCCGCGTTTT |
| Hgf | ATGTGGGGGACCAAACTTCTG | GGATGGCGACATGAAGCAG |
| Egr3 | CCGGTGACCATGAGCAGTTT | TAATGGGCTACCGAGTCGCT |
| Ccn3 | AGTGCCCCAGTATATCACCGA | GTCACAGGGTCTCATCTCAGA |
| Gja5 | CTTCCGCTGTGTGCTACTTGT | GATCCCACGTTGTTGCGTTG |
| Igf1 | CTGGACCAGAGACCCTTTGC | GGACGGGGACTTCTGAGTCTT |
| Edn1 | GCACCGGAGCTGAGAATGG | GTGGCAGAAGTAGACACACTC |
| Gli1 | CCAAGCCAACTTTATGTCAGGG | AGCCCGCTTCTTTGTTAATTTGA |
| Podn1 | GCCCCGAGGAGAATGAATTTG | GAAACTCACGCAGGTCAATGC |
| Egr1 | TCGGCTCCTTTCCTCACTCA | CTCATAGGGTTGTTCGCTCGG |
| Tnfrsf18 | GCCATGCTGTATGGAGTCTCG | CCACTTCCGTTCTGAACCTTG |
| Sema5b | GAAGCCGTGGGTCTTTAACTT | CAAGAGCAAGCTGGGAGAAAT |
| Plvap | GCTGGTACTACCTGCGCTATT | CCTGTGAGGCAGATAGTCCA |
| Pltp | CGCAAAGGGCCACTTTTACTA | GCCCCCATCATATAAGAACCAG |
| Ccl9 | CCCTCTCCTTCCTCATTCTTACA | AGTCTTGAAAGCCCATGTGAAA |
| Rnf166 | AAGGCCACCCATGTAGAGAAG | GAATTTGGGGCAGTTAGCCAT |
| Egr2 | GCCAAGGCCGTAGACAAAATC | CCACTCCGTTCATCTGGTCA |
| Kif5a | ATGGCGGAGACTAACAACGAA | TGGAAAATGGGGATGAACTTGTC |
| Cnn1 | TCTGCACATTTTAACCGAGGTC | GCCAGCTTGTTCTTTACTTCAGC |
| iL11 | TGTTCTCCTAACCCGATCCCT | CAGGAAGCTGCAAAGATCCCA |
| Cd244a | CTCGGGGCCATCATTTGTTTC | GCTAGAAGGGAGCTGAACATCA |
| Adam33 | TGGAGAGCTAGTCACTCCCC | AGGCTTCAAGATCGGCTCCT |
| Chrnb2 | AGGGGTTTTGGGTACTGACAC | AGCTTGTTATAGCGGGAAGGA |
| Rpgrip1 | CTGCCAGTTAGAGACACAGATTC | AGCTCCTTCTGGTTCATCCTG |
| Tnc | ACGGCTACCACAGAAGCTG | ATGGCTGTTGTTGCTATGGCA |
| Dusp15 | GCATGACCAAGGTACTTCCTG | GGGGTGATTCGTGGATAGAGAT |
| Slc25a27 | TCTAACCACTTACGACACAGTGA | GCTTTACCGACCTTCCTTGTTT |
| Epha5 | GTGAAGTTTGCAGTGGATGTTTG | AACGTGTCTAAGGAGCCGTTC |
| Folh1 | TGCTGGCTTTAACCGGAACC | ACCAAATCCAGGCCAAATTCTTT |
| Bmp8b | CCGGGACTCCTATGGCTACT | CATCCGTCATGGCACGGTA |
| Card14 | CAGCCGCATGAAACGTGAG | TCCTTCTCTCTGAGTGCGTTG |
| Eya2 | GCTGACACACACCTGTTCTTC | CTATCAAGCCACCCACGTTGT |
| Gzme | ACCTCCTTCCTCCCCTTCC | CTCCTCTGCTCCAGCTCCA |
| Mcpt8 | CCACTCCCGGCCCTATATG | TGCTGTCATTACGATGTCTCTTG |
| Gdf6 | TATCGCGCCCCTAGAGTACG | ATGCTAATGGGAGTCAGTTTGG |
| Ntf5 | TGAGCTGGCAGTATGCGAC | CAGCGCGTCTCGAAGAAGT |
| Nrbp2 | AGATCCAAGCCAACGGGGATA | GAGCACTCGGTGGAAGAGG |

Data S1.

Uncut Western blots images.
