# Supplementary material for: FSAP aggravated endothelial dysfunction and neurological deficits in acute ischemic stroke due to large vessel occlusion
Source: Signal Transduct Target Ther. 2022 Jan 7;7:6. doi: 10.1038/s41392-021-00802-1 (PMC8738761; doi:10.1038/s41392-021-00802-1)
Supplement: Supplementary file 2 — uncut WB images [file 41392_2021_802_MOESM2_ESM.pdf]

Figure 3

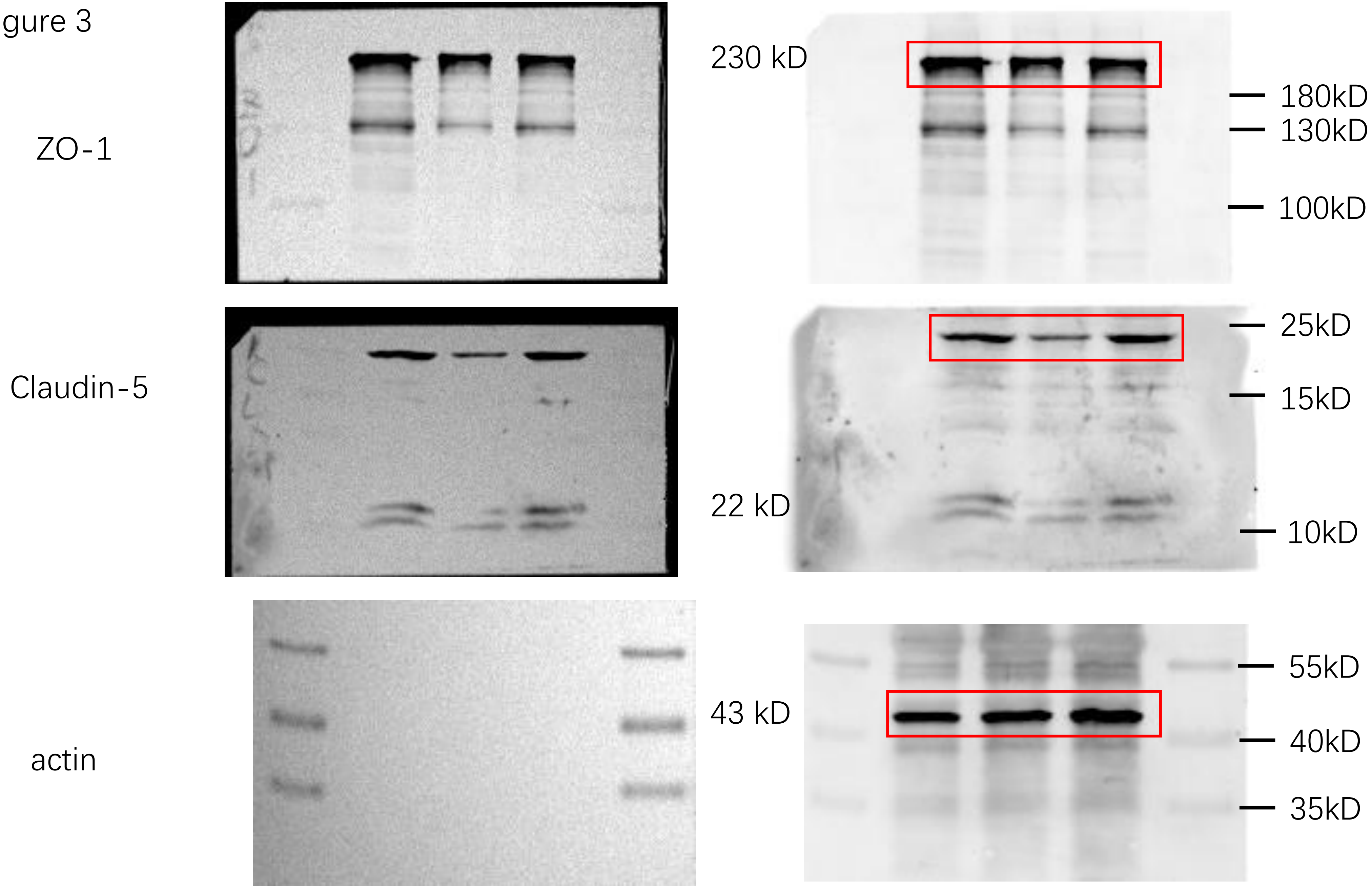

ZO-1
Claudin-5
actin
230 kD
22 kD
43 kD
180kD
130kD
100kD
25kD
15kD
10kD
55kD
40kD
35kD

Figure 4

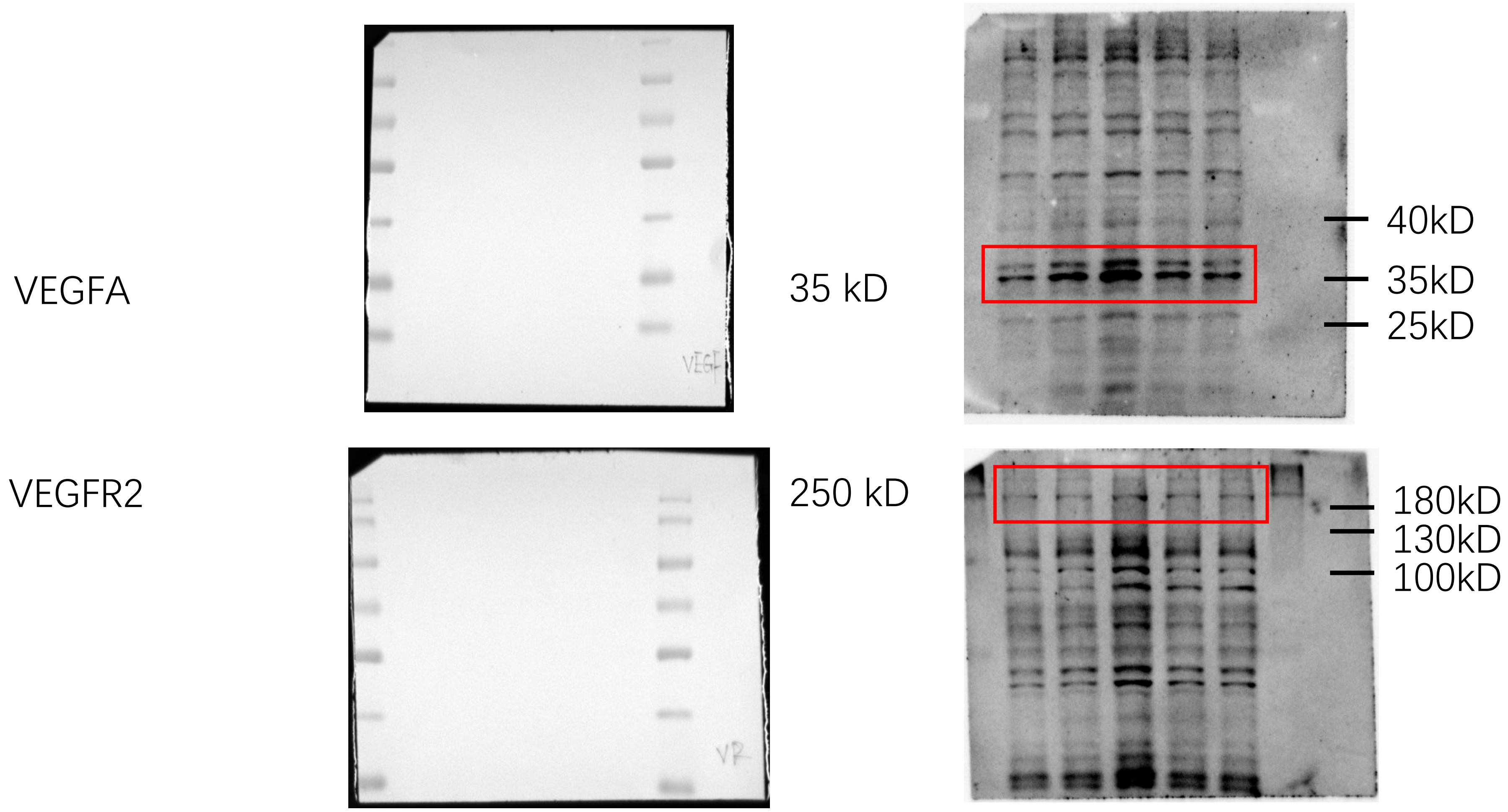

Figure 4

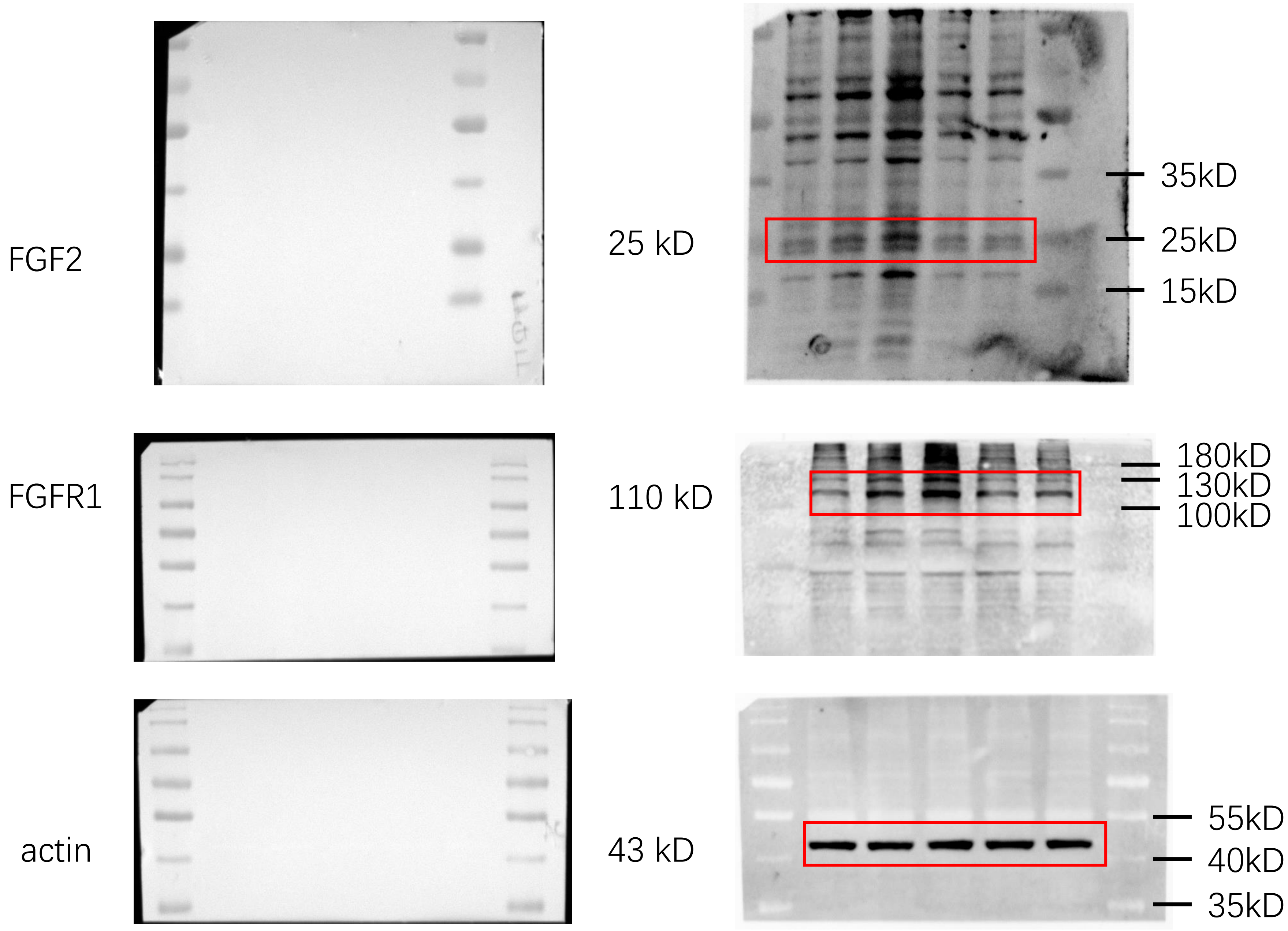
FGF2
25 kD
35kD
25kD
15kD
FGFR1
110 kD
180kD
130kD
100kD
actin
43 kD
55kD
40kD
35kD

Figure S3A

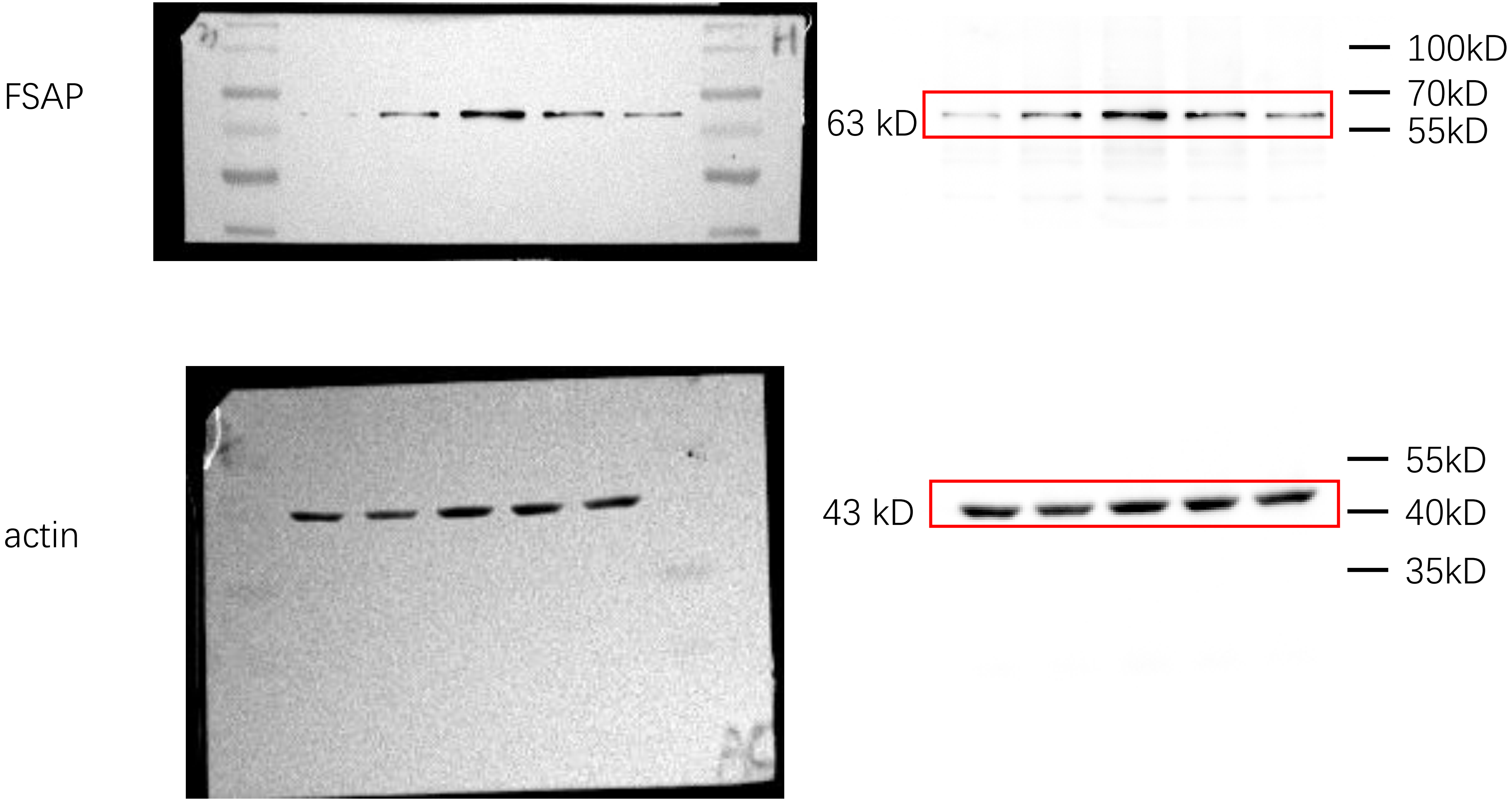

Figure S4A

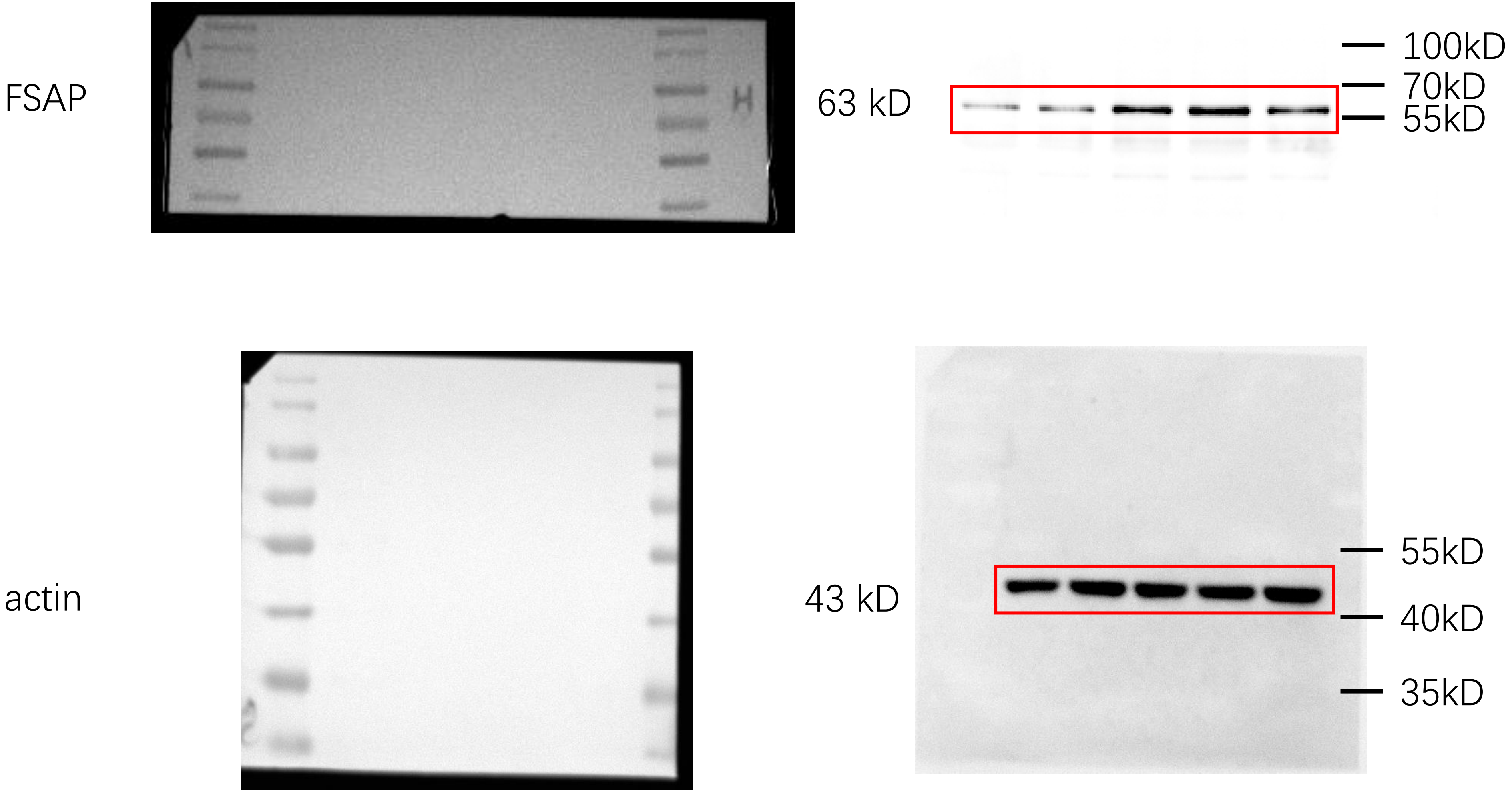

Figure S4A

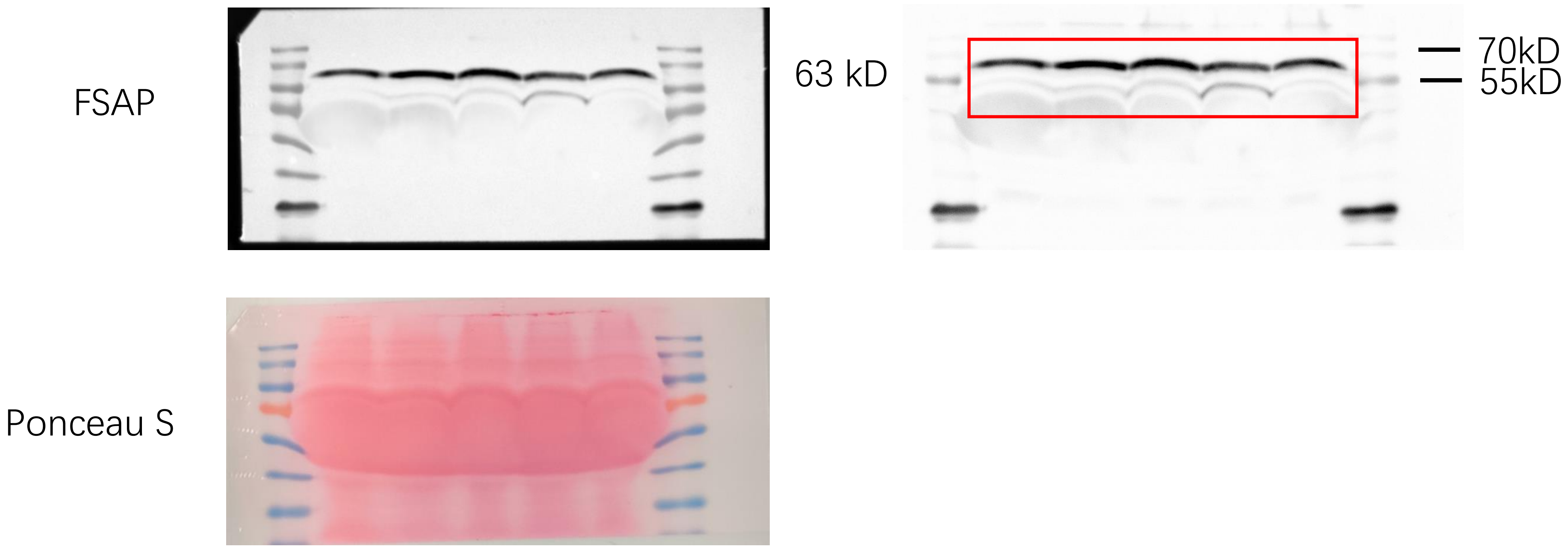

Figure S4B

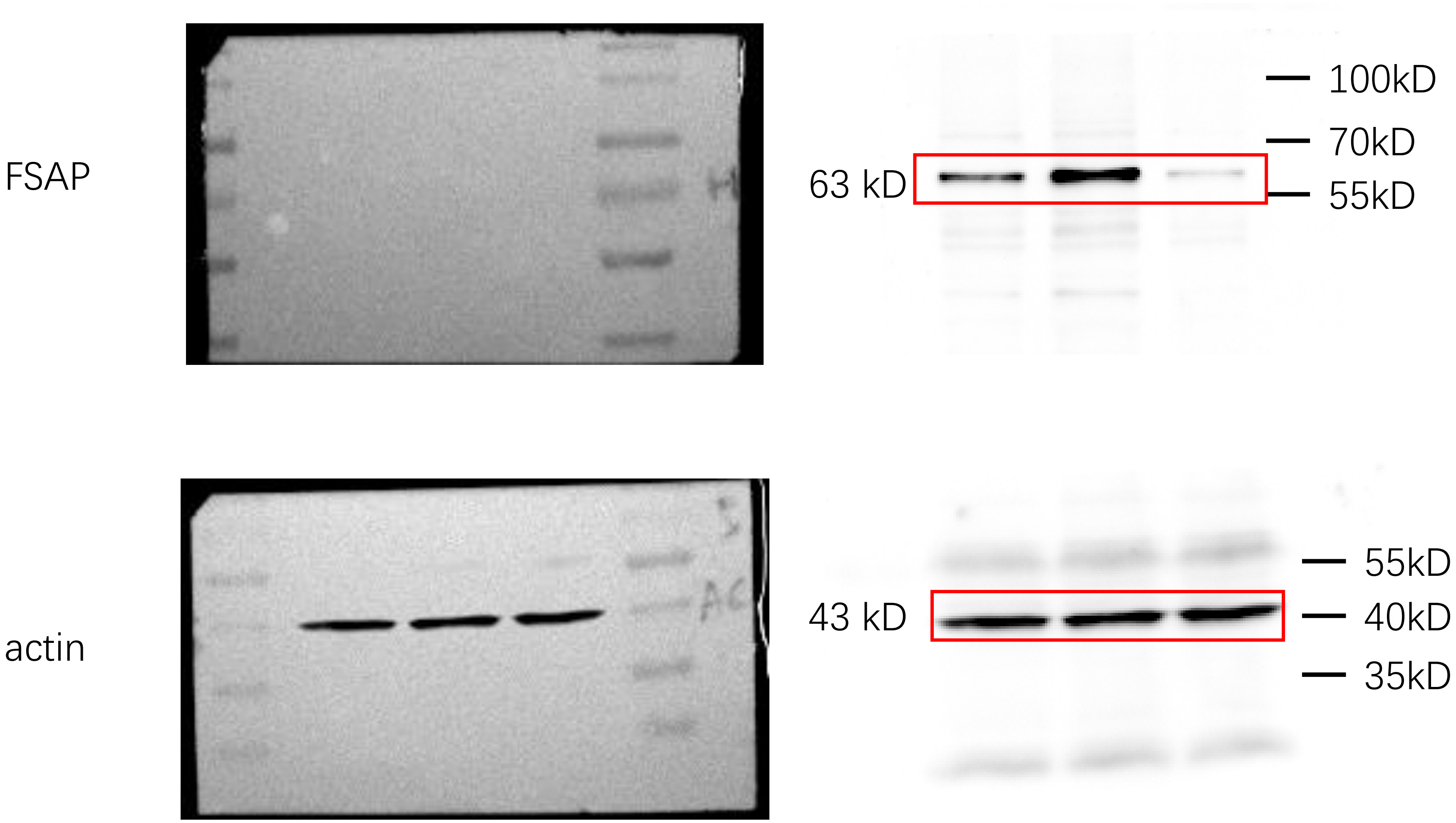

Figure S4B

FSAP

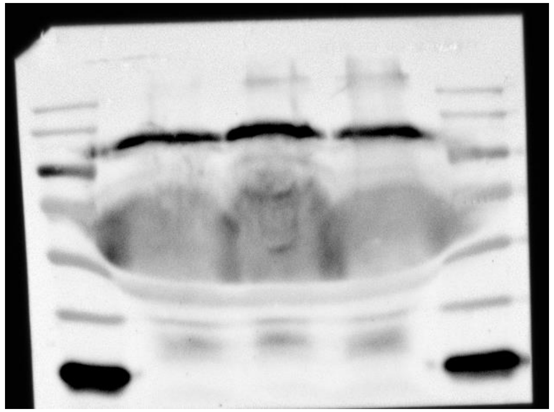

63 kD

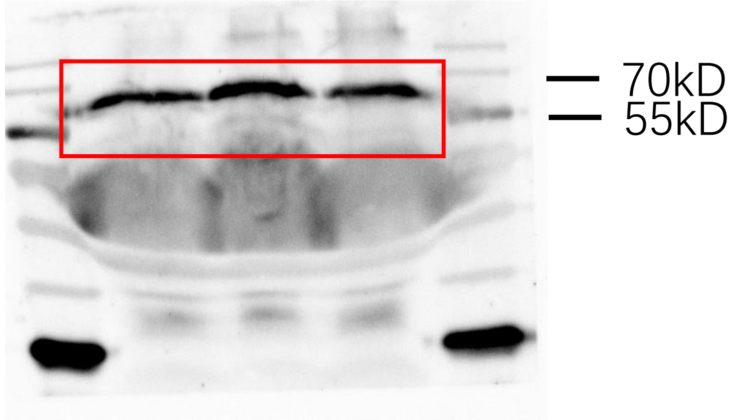

Ponceau S

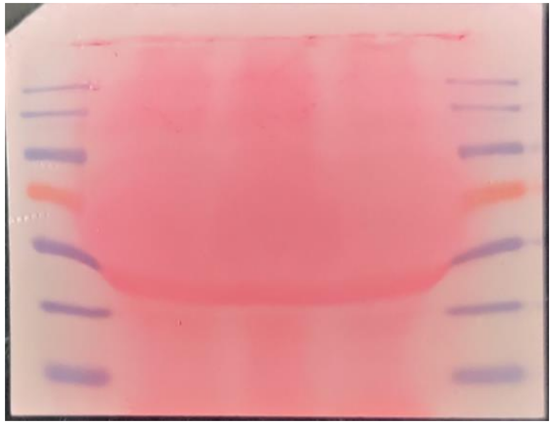

Figure S4E

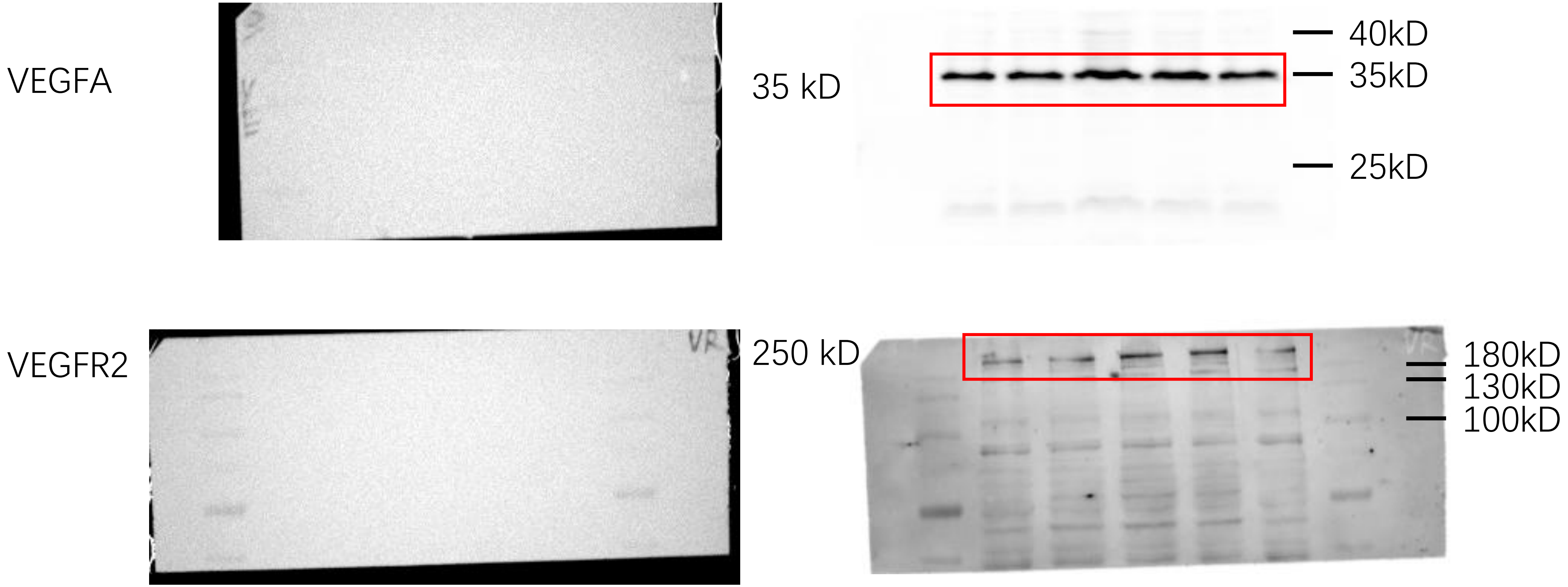

Figure S4E

FGF2

FGFR1

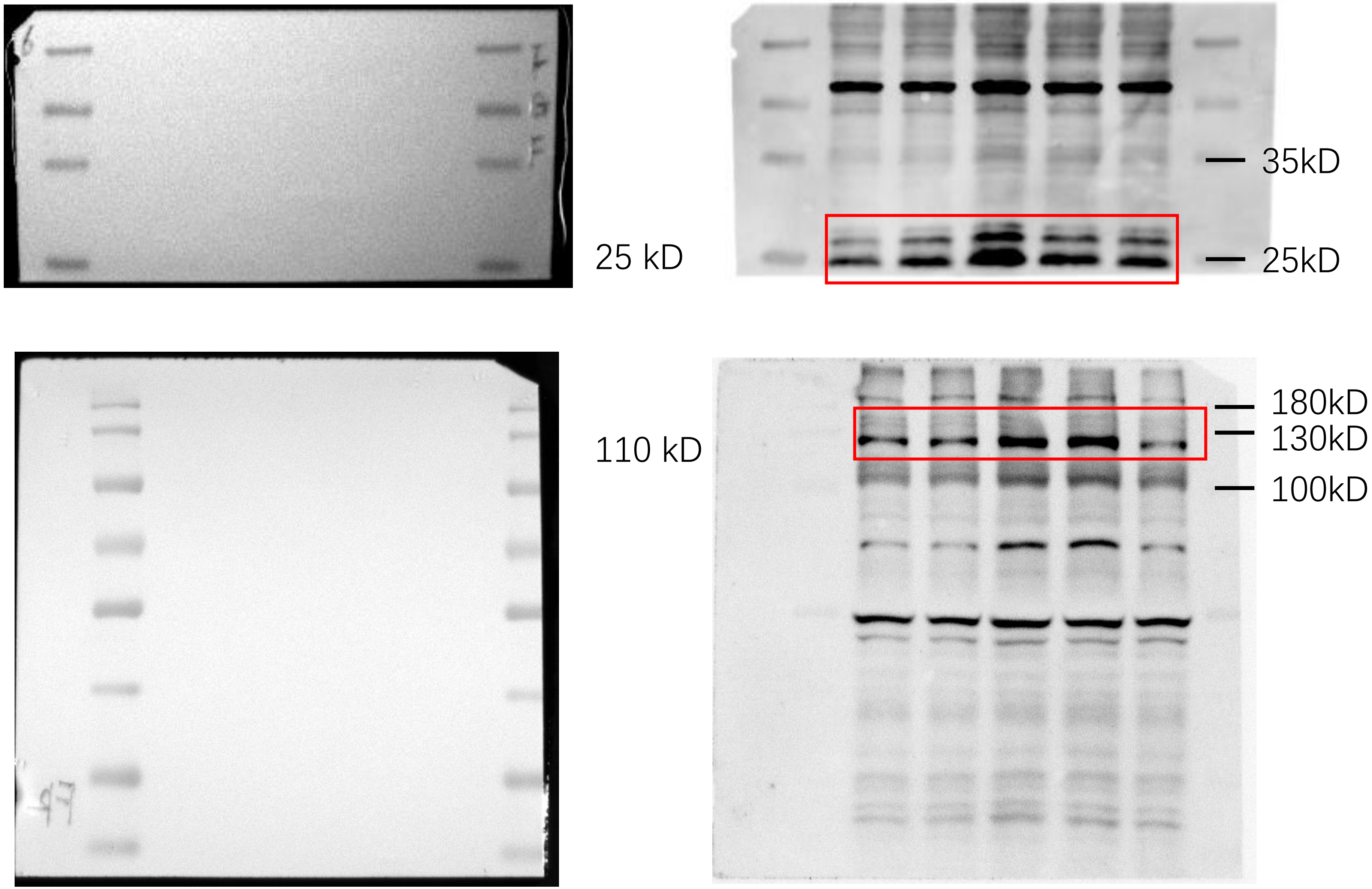

Figure S4E

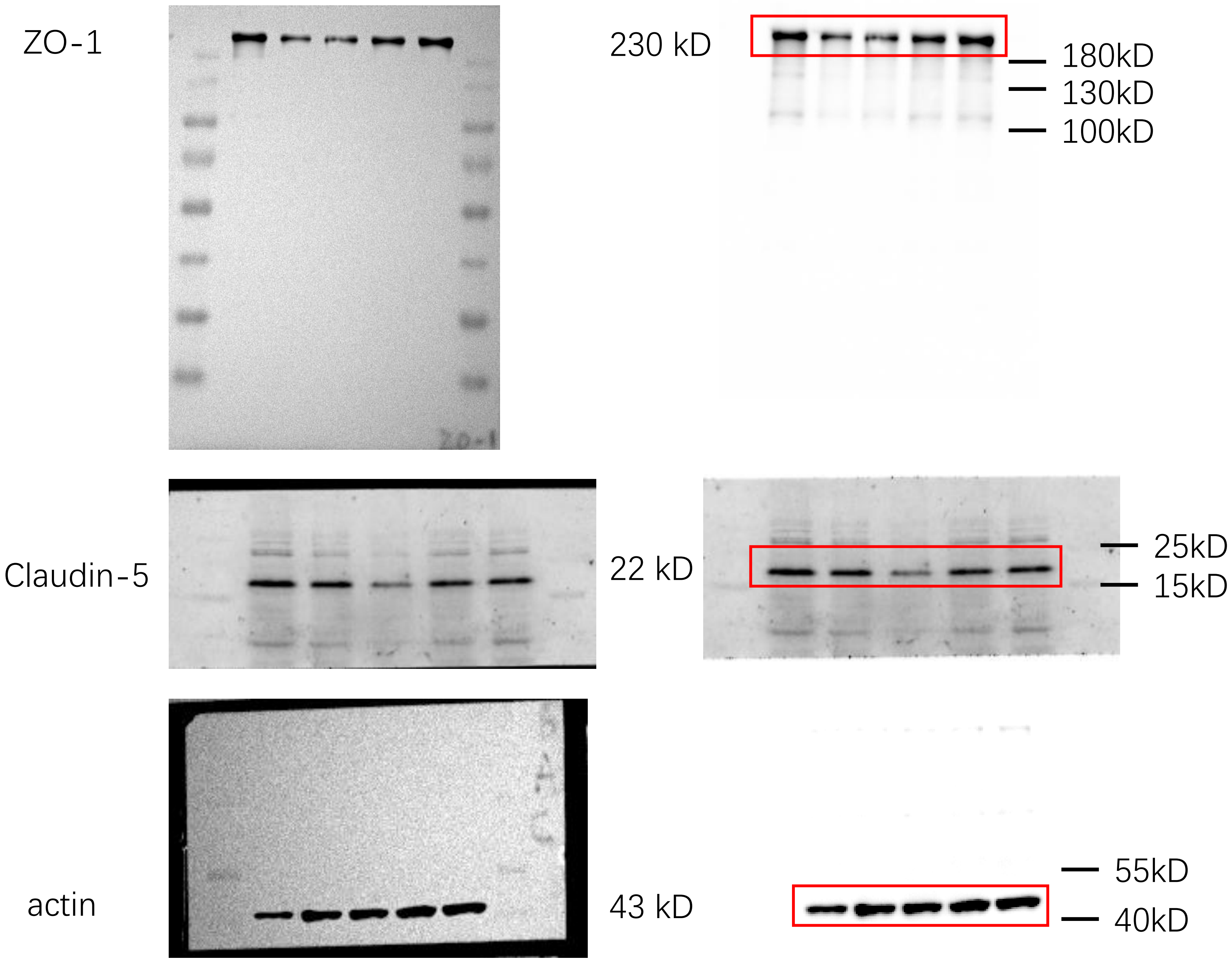
ZO-1
230 kD
180kD
130kD
100kD
Claudin-5
22 kD
25kD
15kD
actin
43 kD
55kD
40kD
